# Functional redundancy in natural pico-phytoplankton communities depends on temperature and biogeography

**DOI:** 10.1098/rsbl.2020.0330

**Published:** 2020-08-19

**Authors:** Duyi Zhong, Luisa Listmann, Maria-Elisabetta Santelia, C-Elisa Schaum

**Affiliations:** 1 Institute for Marine Ecosystem and Fisheries Science, University of Hamburg, 22767 Hamburg, Germany; 2 Centre for Earth System Science and Sustainability, 20146 Hamburg, Germany

**Keywords:** functional redundancy, global warming, pico-phytoplankton communities, Baltic Sea

## Abstract

Biodiversity affects ecosystem function, and how this relationship will change in a warming world is a major and well-examined question in ecology. Yet, it remains understudied for pico-phytoplankton communities, which contribute to carbon cycles and aquatic food webs year-round. Observational studies show a link between phytoplankton community diversity and ecosystem stability, but there is only scarce causal or empirical evidence. Here, we sampled phytoplankton communities from two geographically related regions with distinct thermal and biological properties in the Southern Baltic Sea and carried out a series of dilution/regrowth experiments across three assay temperatures. This allowed us to investigate the effects of loss of rare taxa and establish causal links in natural communities between species richness and several ecologically relevant traits (e.g. size, biomass production, and oxygen production), depending on sampling location and assay temperature. We found that the samples' biogeographical origin determined whether and how functional redundancy changed as a function of temperature for all traits under investigation. Samples obtained from the slightly warmer and more thermally variable regions showed overall high functional redundancy. Samples from the slightly cooler, less variable, stations showed little functional redundancy, i.e. function decreased when species were lost from the community. The differences between regions were more pronounced at elevated assay temperatures. Our results imply that the importance of rare species and the amount of species required to maintain ecosystem function even under short-term warming may differ drastically even within geographically closely related regions of the same ecosystem.

## 1. Introduction

When ecosystems lose species, the function and services of these ecosystems may decline. Evidence has long been emerging for this to be true across taxa and biomes, spanning heterotrophic and autotrophic microbial communities to plants to metazoans, i.e. ecosystems with higher biodiversity tend to be more productive on average regardless of the type of organism under investigation [1–5]. In a warming world, variation in temperature may shape the biodiversity–ecosystem function relationship on short (days to weeks) and long-term timescales (years to decades and beyond) [6,7]. A recent study has experimentally examined the synergistic effects of warming and biodiversity loss on function in bacterial assemblages [8], but no comparable data exist for natural phytoplankton communities. As a consequence, studies on phytoplankton biodiversity and ecosystem function remain largely observational [1] and usually do not consider loss of biodiversity in interaction with aspects of climate change (but see [9]) or evolutionary history (but see [10] for population genetics). Pico-phytoplankton contribute about 20% of phytoplankton primary production [11], and unlike larger phytoplankton, they do not form blooms, but contribute to the photosynthesizing foundation of aquatic ecosystems year round [12,13]. They show rapid physiological [14,15] and evolutionary [16,17] responses to changing environments when studied as single-strain cultures. There is isolated evidence that the speed and mechanism of evolutionary responses in a changing environment may differ between phytoplankton strains evolving in isolation and strains evolving in communities, mixed cultures or multi-genotype biofilms [18–22]. Comparable experiments have not been carried out for pico-phytoplankton (but see [23]). As a consequence, we need to conduct manipulative experiments to establish a better understanding of how the links between pico-phytoplankton community diversity and pico-phytoplankton community function change with temperature on short- and long-term timescales. Since natural phytoplankton strains are notoriously difficult to grow in isolation under laboratory conditions, assembling artificial communities from natural phytoplankton components can be an arduous task. Organisms from culture collections which have already been shown to grow well in isolation are easier to assemble into communities (e.g. [9]) but may not reflect well the complex environments that the lineages were originally sampled from. Therefore, when working with natural assemblages, serial dilution of natural samples can provide a useful tool to reduce biodiversity to such an effect that rare species are lost, and common species remain [24,25].

**Figure 1.**
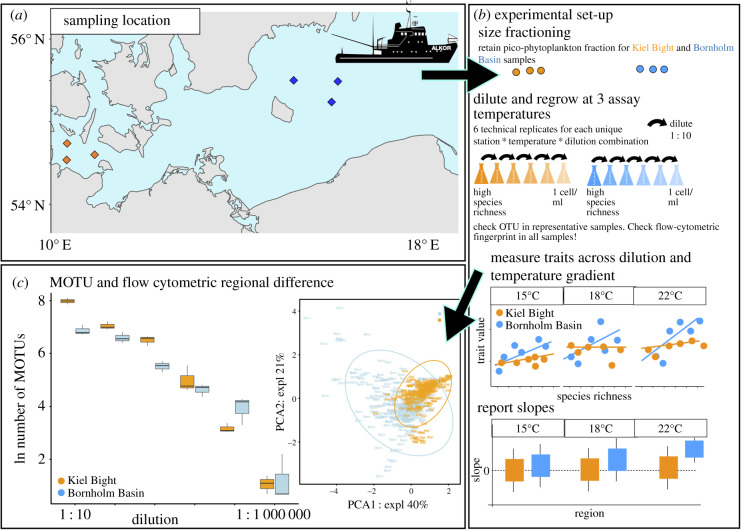
Overview of sampling locations, experimental set-up and assessment of species richness via meta-barcoding and phenotypic composition via flow cytometry. (*a*) Sampling locations: we took community samples in two biogeographically distinct regions of the Southern Baltic Sea: the Kiel Bight (KB, orange squares), and the Bornholm Basin (BB, blue squares) (electronic supplementary material, table S2). (*b*) Set-up and data-processing: the pico-phytoplankton fraction was obtained through size fractioning on board. Upon returning to the Institute for Marine Ecosystem and Fishery Science Hamburg (IMF), we established six technical replicates per community for each dilution across the three assay temperatures (15°C, 18°C and 22°C). We report the slopes of the traits across the dilution steps for each region*assay temperature combination. (*c*) Proof of concept via meta-barcoding and flow cytometry: to test our experimental design, we obtained MOTUs (meta-barcoding operational taxonomic units) and regional differences between samples based on flow-cytometric data (based on size and photosynthetic pigment composition). While there were some regional differences in initial MOTU composition, those were overall not significant. (See also electronic supplementary material, figures S5 and S7 for higher resolution images of (*c*), and electronic supplementary material, S6–S11 for flow-cytometric characteristics and diversity.)

Here, we investigated the combined effects of short-term warming and biogeographic history on the diversity–function relationship in natural pico-phytoplankton communities. To do so, we obtained pico-phytoplankton community samples during two cruises of RV ALKOR (AL505 and AL513) on the Southern Baltic Sea in 2018 (electronic supplementary material, tables S1 and S2). Of the two basins sampled, the Kiel Bight is characterized by on average 2°C higher temperature than the Bornholm Basin and unpredictable fluctuations in temperature on the timescale of days to months (electronic supplementary material, table S3). In the Bornholm Basin, fluctuations in temperature follow a highly predictable pattern governed by seasonality (electronic supplementary material, table S3). The basins are connected through currents [26], and the regions are geographically close to each other (figure 1) so that we can rule out confounding effects introduced by e.g. differences in precipitation, day length and light intensity [27]. By assaying the effect of species loss on communities across three temperatures within the range of Southern Baltic Sea spring and summer temperatures [26,27], we can test the contributions of long-term (comparison of basins) and short-term (assay temperatures) changes in temperature on key community functions.

## 2. Summary of methods

The full methods, especially concerning our rationale for the culture temperatures and common garden approach chosen and the calculations/cytometer settings for phenotypic diversity, are available in the supporting information.

We obtained pico-phytoplankton community samples during two RV ALKOR cruises (AL505 and AL513 respectively) in 2018 (figure 1; electronic supplementary material tables S1 and S2 for sampling dates and locations) from 5 m. Samples were size fractioned to obtain the pico-phytoplankton community. To rule out effects of parameters other than temperature and diversity during the experiment, all samples were grown in f/2 media [28] at the salinity of the sampling location on board and in the laboratory. In the laboratory, community samples first grew in semi-continuous batch culture at 100 µmol quanta m^−2^ s^−1^ (12 : 12 light/dark cycle) at 15°C for samples from AL505 and 22°C for samples from AL513 (11 and 7 months, respectively, with fortnightly growth rate and community composition measurements). They were then transferred to a common garden at 18°C, where they were batch-cultured until used for the experiment (detailed timeline electronic supplementary material, table S1). To seed the dilutions, we counted cell numbers in all samples on a BD Accuri C6 flow cytometer. The flow cytometric fingerprints also allow for an estimate of phenotypic diversity or trait-level diversity [29,30] (electronic supplementary material, figures S7–S11). Samples were diluted in six 10-fold dilution steps at the appropriate salinity, down to the lowest point of dilution (in theory containing no more than one species or pico-phyoplankton per millilitre). Six technical replicates of each sample were left to regrow to 10^6^ cells ml^−1^ at the assay temperatures of 15°C, 18°C and 22°C. We re-diluted all samples to 3000 cell ml^−1^ for tracking growth curves. Cell size was obtained from the flow cytometer to calculate an estimate of picograms of carbon per millilitre after [31].

Net photosynthesis rates were obtained when samples were in exponential phase, on PreSens^®^ SDR Sensor Dish optodes. We measured oxygen production for 15 min in the light and respiration for 15 min in the dark.

We obtained two measures of biodiversity in our samples. One, following CTAB DNA extractions [32], samples were sent for DNA meta-barcoding at biome-id, resulting in a MOTU (meta-barcoding operational taxonomic units) estimate for those samples. Two, phenotypic diversity [29,30,33] was assessed using the parameters returned by the flow cytometer.

## 3. Statistical analysis

All data were analysed in the R programming environment (v. 3.5.3.). To analyse the shape of the growth curves, nonlinear curve fitting of a Baranyi growth model [34] was carried out using the ‘nlsLM’ function in the R package, ‘minpack.lm’. For multi-model selection, we computed small sample-size corrected AIC scores (AICc) and then compared the models by calculating delta AICc values and AICc weights using the ‘MuMIn’ package. We used the PhenoFlow package [30] for the calculation of phenotypic diversity. Simplified F statistics are in electronic supplementary material, table S6. Detailed model selection and output in electronic supplementary material, tables S7–S9.

## 4. Results

### (a) Proof of concept

MOTU analyses revealed that biodiversity (as species richness) in undiluted samples was on average 1.1-fold higher for the Kiel Bight than the Bornholm Basin sampling region (figure 1*c*; electronic supplementary material, figure S5) and roughly in line with previous studies on Southern Baltic Sea phytoplankton communities [35]. MOTU diversity scaled with phenotypic diversity (electronic supplementary material, figure S2). Diversity, once established by dilution, did not change significantly throughout the time of culture (electronic supplementary material, figures S8–S9) or the growth curve (electronic supplementary material, figures S10–S11). The latter matters here as we report net photosynthesis during exponential growth, but other parameters, at carrying capacity.

### (b) Assay temperature and biogeography explain differences in functional redundancy

**Figure 2.**
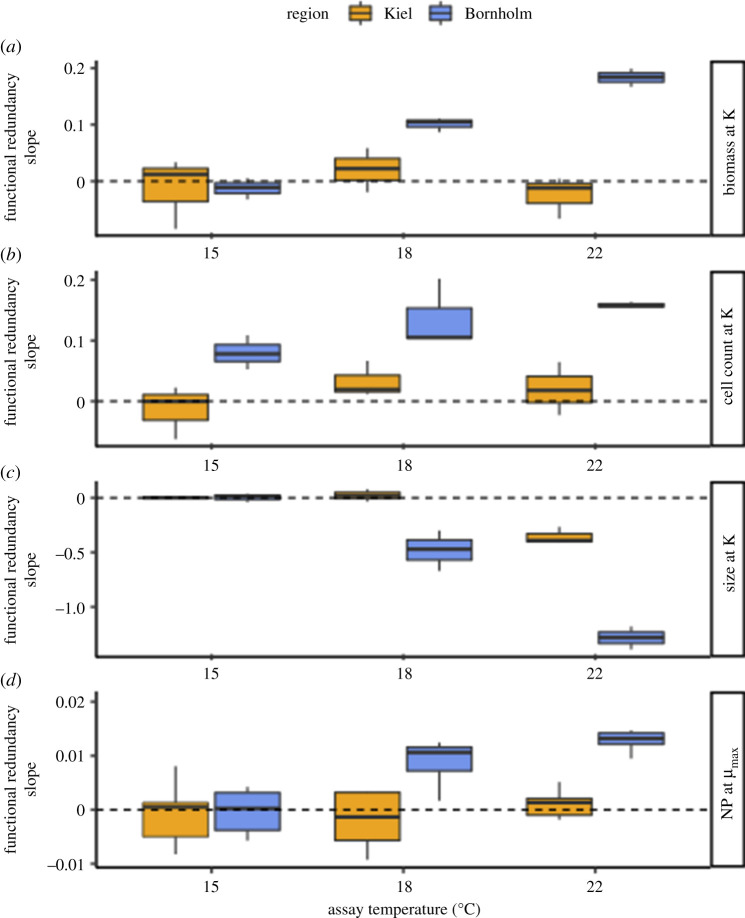
Functional redundancy as steepness of slopes for traits across dilution gradients (electronic supplementary material, figures S12–S16, electronic supplementary material, table S5 for slopes). The dashed line shows a slope of 0, indicating high functional redundancy, i.e. the trait does not change significantly as rare species are lost; deviations from 0 are not in line with functional redundancy. (*a*) Biomass produced at carrying capacity (K) is stable across dilution steps and temperatures for samples from the Kiel Bight (high functional redundancy); however, there is low functional redundancy in samples from the Bornholm Basin, especially under warming. (*b*) Cell count (ml^−1^) at carrying capacity. Cell count followed the same pattern as overall biomass. (*c*) Cell size at carrying capacity. Average cell diameters were independent from dilution at 15°C for samples from either region, but cells became smaller faster with temperature in the full communities as compared to low species richness samples. This effect was more pronounced for samples from the Bornholm Basin. (*d*) Net photosynthesis (NP) across dilution steps during exponential growth (µ) showed similar patterns as the biomass response. Orange for Kiel Bight samples, blue for Bornholm Basin samples. The boxplots are displayed as is standard, with the girdle band indicating the median, and the whiskers extending to the 25th and 75th percentile. *n* = 6 for each unique combination. This plot only displays significant parameters, i.e. samples from different seasons have been pooled.

We consider a result to be in line with high functional redundancy, when a function—such as biomass production or net photosynthesis—does not change significantly across levels of species richness. This results in slopes across dilution steps (i.e. species richness) that are equal or close to zero. When a function declines with species richness, we assume low functional redundancy. In these cases, the slope of trait value as a function of species richness is usually *positive* [24]*.* A *negative* slope is also a deviation from zero and therefore from functional redundancy in *sensu stricto*; however, depending on the trait in question, a negative slope may not always be associated with a loss in function. We report the steepness of these slopes across three assay temperatures in figure 2 (electronic supplementary material, table S5).

Across dilutions, temperature had a significant impact on biomass (figure 2*a*; electronic supplementary material, figure S12, likelihood ratio test comparing models with and without ‘temperature’: Δd.f. = 2, *χ*^2^ = 382.71, *p* < 0.0001). When communities lost rare species, biomass production did not change significantly in the samples from the Kiel Bight. Biomass production in samples from the Bornholm Basin samples rapidly decreased when rare species were lost, resulting in positive slopes (likelihood ratio test comparing models with and without ‘region’: Δd.f. = 2, *χ*^2^ = 185.23, *p* < 0.0001).

Changes in biomass were driven by a change in cell number and a change in cell volume across dilution steps, with a trend for smaller cells at higher species richness (figure 2*b,c*; electronic supplementary material, figures S13–S14). Cell volume decreased with temperature (likelihood ratio test comparing models with and without ‘temperature’: Δd.f. = 2, *χ*^2^ = 126.95, *p* < 0.0001). While cells in samples from the cooler, more stable, Bornholm Basin were on average less reactive to temperature in terms of cell size, the effect of losing rare species was stronger here than in samples from the Kiel Bight and cell size rapidly increased as species richness decreased (likelihood ratio test comparing models with and without ‘region: Δd.f. = 2, *χ*^2^ = 165.10, *p* < 0.0001).

Net photosynthesis rates (NP) per cell followed a similar trend to biomass (figure 2*d*; figures S15–S16): assay temperature altered community NP depending on dilution and sampling origin. Samples from the Kiel region were overall more photosynthetically active than those from the Bornholm region (likelihood ratio test comparing models with and without ‘region’: Δd.f. = 3, *χ*^2^ = 45.63, *p* < 0.0001), especially at the warmer temperatures (likelihood ratio test comparing models with and without ‘temperature’: Δd.f. = 1, *χ*^2^ = 41.47, *p* < 0.0001). NP in samples from the Kiel region showed no clear trend with dilution. In samples from the Bornholm region, on the other hand, responses of NP to temperature were overall less pronounced, but were strongly influenced by species richness, with the full community samples photosynthesizing nearly 1.5 times (per cell) as much as the samples with the lowest species richness.

## 5. Discussion

Our investigations of the link between species richness and traits relevant for ecosystem services showed that the steepness of the biodiversity–function slope hinged on the communities' evolutionary histories as well as on short-term (*ca* 14–20 generations) changes in temperature and fit well within the theoretical and observational general framework of biodiversity/ecosystem function studies [1–4]. The effect of temperature modulating the strength of the diversity/function relationship was the most pronounced for samples originating from a cooler, less variable region, in line with theory predicting that regions that are more variable should contain a greater number of taxa with more variable tolerance thresholds [36]. Our results highlight the importance of long-term contributions of the thermal peculiarities in the two basins. We argue that thermal adaptation to an overall warmer, more variable basin may mitigate short-term responses to temperature.

Overall, our findings are in accordance with the literature on experimentally assembled communities in microcosms, where the researchers found that a decline in productivity was more pronounced at rising temperatures in phytoplankton and bacteria ([9] and [8], respectively). We make the case that this pattern of declining productivity due to species loss being enhanced at higher temperatures may be conserved across environments as diverse as laboratory conditions and natural community samples. This may also mean that results obtained from assembling artificial communities reflect results found in natural assemblages well in direction and possibly in magnitudes of responses. We specifically find that as species are lost from samples with a history of comparatively cooler average temperatures and highly predictable seasonal variation, the community rapidly shows a decrease in net photosynthesis rates, biomass production and an increase in cell size. We can only speculate about the underlying mechanisms. However, we can rule out that competition for macro-nutrients, light or space were the sole drivers due to the replete culture conditions. Neither can the pattern be fully explained by loss of large cells through dilutions (this would have been obvious in the flow cytometer data), or the prevalence of small cyanobacteria in the Bornholm summer samples. The pattern may be driven in part by competition for micronutrients not commonly added to f/2 medium, and by interactions beyond competition, e.g. types of facilitation [37], or a more general trend for trait variability and biodiversity being intertwined [38]. While there are clear connotations for loss of biomass and low photosynthesis rates, the implications of changes in cell size on, for example, food-web function and carbon export, are much less clear and cannot directly be regarded as a loss of ecosystem function.

Biodiversity manipulation by dilution has limitations (e.g. diversity and species identity can never be fully disentangled, quasi-random differences in beta diversity), but these apparent disadvantages allow us to specifically investigate the importance of rare taxa, rather than random species loss. Our study combines marine biology, ecology and evolution and is to our knowledge the first of its kind. As such, it has not been without pitfalls. Although we still found that the region of origin, not the culturing temperatures, robustly explained our results best, we recommend that future studies aim to reduce the time that communities are kept in the laboratory prior to the measurements. Where sampling region has a direct impact on growth rates regardless of other assay conditions, it is crucial to consider whether prolonged laboratory exposure of faster growing lineages leads to greater laboratory effects than in slower growing lineages. A more in-depth analysis focused on meta-barcoding will aid in getting a better picture of species identities throughout the experiment. Further, the Baltic Sea is generally low in species due to its characteristically brackish waters. To rule out that our results are typical for regions where diversity is low to begin with, it would be necessary to carry out studies across systems that vary in their initial species compositions. It will be desirable to identify similar systems world-wide, for example within the Mediterranean, connected geothermal lake systems, or coastal versus off-shore systems. Together, such data will further improve our understanding of the relationship between diversity and ecosystem function at the foundation of warming aquatic ecosystems.

## Supplementary Material

Main Supporting Information File

## Data accessibility

Data are available in the supporting information, and https://dx.doi.org/10.5061/dryad.0p2ngf1xw [39].
