# Supplementary material for: Functional redundancy in natural pico-phytoplankton communities depends on temperature and biogeography
Source: Biol Lett. 2020 Aug 19;16(8):20200330. doi: 10.1098/rsbl.2020.0330 (PMC7480144; doi:10.1098/rsbl.2020.0330)
Supplement: Main Supporting Information File [file rsbl20200330supp1.pdf]

Supporting information for

Functional redundancy in natural phytoplankton communities depends on temperature and biogeography

Contributing authors:
Duyi Zhong[1], Luisa Listmann[1,2], Maria-Elisabetta Santelia[1,2], C-Elisa Schaum[1,2]

Author affiliations:
[1] University of Hamburg, Institute for Marine Ecosystem and Fisheries Science, 22767 Hamburg
[2] Centre for Earth System Science and Sustainability, 20146 Hamburg

Corresponding author
elisa.schaum@uni-hamburg.de

Table of Contents

Full methods ... 3
Table S1: Time-line of experiment, ... 8
Table S2: Sampling station overview ... 9
Figure S1: Thresholded fraction containing debris, as well as bacterial (E4), viral (R3), and assumedly pico-eukaryotic matter ... 13
Figure S2: An example of FL3 (PerCP) against FSC with a fairly high threshold of 2500 on FSC and FL3 ... 14
Figure S3: An example cytogram for SSC, FSC, FL2, FL3, and FL4 in one of the high diversity experimental units. ... 15
Figure S4: An example cytogram for SSC, FSC, FL2, FL3, and FL4 in one of the low diversity experimental units. ... 16
Statistical analysis ... 18
Further Supporting Figures ... 20
Figure S5: Dilution is strongly correlated to MOTU (operational taxonomic units obtained through meta barcoding) ... 20
Figure S6: Phenotypic diversity scales with MOTU diversity ... 21
Figure S7: Phenotypic characteristics for Kiel Bight and Bornholm Basin samples did not differ significantly after time in the common garden ... 22
Figure S8: Phenotypic characteristics for Kiel Bight and Bornholm Basin remained largely stable between incubation at 15ºC or 22ºC and the common garden period, but differed slightly from t0 ... 24
Figure S9: Phenotypic diversity for Kiel Bight and Bornholm at t0, incubation at 15ºC or 22ºC and the common garden period at 18ºC ... 26
Figure S10: Once established through dilution, community phenotypic characteristics remained largely stable throughout the growth cycle. ... 28
Figure S11: Once established through dilution, community phenotypic diversity also remained largely stable throughout the growth cycle. ... 29
Figure S12: Biomass at carrying capacity K ... 30
Figure S13: Cell count at carrying capacity ... 32
Figure S14: Size (diameter in µm) at carrying capacity as used for biomass estimation ... 33

Figure S15 Rates of net photosynthesis (µmol O_2 per cell and hour) **during exponential growth** 34
Figure S16 Rates of net photosynthesis (LOG10 µmol O_2 per cell and hour) during exponential growth 35
Figure S17: Growth rates (at µmax) 36

Further Supporting Tables 37
Table S3: Decomposition analysis for estimates of environmental fluctuations 37
Table S4: Nomenclature for flow cytometry parameters 37
Table S5: Slopes obtained (per station) from Figures S12 to S16 37
Table S6: Summary F statistics: 39
Table S7: Model selection (A) output (B) for investigating the effect of dilution (abbreviated to D), assay temperature (abbreviated T), region (abbreviated R), and season (abbreviated S) on biomass produced at carrying capacity 41
Table S8: Model selection (A) output (B) for investigating the effect of dilution (abbreviated to D), assay temperature (abbreviated T), region (abbreviated R), and season (abbreviated S) on cell diameter (µm) at carrying capacity 44
Table S9: Model selection (A) output (B) for investigating the effect of dilution (abbreviated to D), assay temperature (abbreviated T), region (abbreviated R), and season (abbreviated S) on Net Photosynthesis rates (µmol O_2 per cell and hour - displayed as LOG10 values for brevity) 47

Supporting References 49

References for R packages used 50

Full methods

Sampling and on-board treatment and husbandry

We obtained pico-phytoplankton community samples during two RV ALKOR cruises (AL505 and AL513 respectively) in 2018 (see Table S1 for a time line, Figure 1 and Table S2 for sampling dates and locations, Table S3 for decomposition analysis output regarding the environmental fluctuations characterising the sampling regions) using a Niskin bottle at 5m. The Niskin bottle was solitary, and dispatched via a controlled crane. As the CTD data revealed that surface waters were fully mixed, one Niskin-sample of 10L was taken per station. As the Baltic Sea is higher in biomass than for example oligotrophic ocean waters, we found that of these, 2L sufficed for all experiments that followed. Water from each station was immediately passed through a 35µm sieve to remove grazers and large debris, and then further size fractioned *via* gentle filtration with a vacuum pump at the lowest setting. During filtration, we first passed the water sample through a 2µm membrane filter (kept filtrate) to remove organisms larger than the picoplankton fraction, and then an 0.2µm filter. On the 0.2µm filter, we concentrated the 2L-filtrate to an end volume of 200mL. Great care was taken to not let the filter never fall dry to ensure that cells did not get stuck in or were damaged by the pores on the membrane. The filter was rinsed gently with the remaining water above the filter such that the organisms were continuously more concentrated. 30mL of the 0.2µm filtrate were frozen for nutrient analyses in technical duplicates. We used Whatman-Nuclepore polycarbonate track-etched membrane filters with a size of 47mm for all filtration processes.

Throughout the cruise, acute thermal profiles of photosynthesis and respiration for the communities were determined during on-board incubations in order to better be able to estimate which temperatures to use as assay temperatures in the laboratory. Time taken for sample preparation (filtration, incubation of samples in the dark prior to photosynthesis

measurements) is on the scale of hours. The measurement of a full photosynthesis-irradiance curve on an oxygen electrode takes about 20 minutes, including a dark phase for respiration. As such, we can be fairly certain that our measurements tracked responses to temperature within the same generation.

On board, an aliquot of each community was immediately frozen in sorbitol for later (upon return to Hamburg) analysis on the flow cytometer.

All communities were transferred into full f/2 media [1] at the salinity of the sampling location to rule out effects of parameters other than temperature and diversity during the experiment. AL505 samples from an in-situ temperature of 1-2ºC were first stored at 4ºC for 24-48 hours, and then in a 10ºC cold storage room on board for the remainder of the cruise (2-12 days depending on cruise and day of sampling). We used LED light stripes for an irradiance of approximately 100 µmol quanta m^{-2} s^{-1}, at a 12h/12h light/dark cycle. Irradiance in Baltic Sea surface waters can fluctuate dramatically (between 30 µmol quanta m^{-2} s^{-1} and 3000 µmol quanta m^{-2} s^{-1}) within even a day. 100 µmol quanta m^{-2} s^{-1} was found to be a light-intensity suitable for culture under the conditions on board and in our laboratory. We suggest that for similar studies, each researcher carry out pilot experiments to establish the appropriate light levels.

AL513 samples from an *in-situ* temperature of 21ºC to 23ºC were also stored in the cold room (at 10ºC) using the irradiance and media conditions above. We have found that this does not 'shock' the samples, but puts them into a gentle stasis until further use, so as long as the period at colder temperatures does not exceed 2-3 weeks.

<u>Treatment and husbandry of communities in the laboratory</u>

As during the time on board, to rule out effects of parameters other than temperature and diversity during the experiment, all samples were grown in f/2 media [1] at the salinity of the

sampling location. Community samples grew in semi-continuous batch culture at 100 µmol quanta m^{-2} s^{-1} (12:12 light/dark cycle) in 40mL of media using vented-cap bent-neck, fully transparent Nunclon® flasks. Batch-transfers occurred fortnightly, and at least at these (sometimes in between) detailed cytograms were taken to track community composition (see below for details on flow cytometry). Communities from AL505 were kept at 15ºC for 11 months. Communities from AL513 were kept at 22ºC for 7 months.

<u>Rationale for culturing temperatures in the laboratory until start of experiment</u>

We had to walk a very fine line between multiple requirements (not all of them in our hands).: i) biomass in the samples was, while not too low for metabolic measurements, too low for MOTU analysis, so a growing period would have been necessary in any case (where time, space, and logistics allow, filtration of much larger samples may also help) ii) this project was carried out as part of an MSc/MRes thesis. To make sure that we stayed within a range of parameters that allows for good growth and a time-frame that is manageable for such a project, we had to choose a higher temperature than was found *in situ* at time of sampling (growth of the community samples even at 5ºC-8ºC is extremely slow and the experiment would have taken a year, and net photosynthesis rates are near the detection limit at these temperatures), iii) 1-2ºC are not a common temperature for the Southern Baltic Sea in March. Water temperatures between 4ºC and the low double digits are much more common (we sampled during an unexpected cold snap). The photosynthesis measurements carried out on board indicated that samples did best at temperatures exceeding 15ºC. This is not surprising given the usual spring temperatures in the Baltic Sea, and a tendency for ectotherms to have their thermal optima slightly above usual environmental levels [2].

Nevertheless, we took great care that AL505 communities were *gradually* transferred to warmer temperatures (see above)

For communities retrieved during AL513, we chose an incubator temperature of 22ºC based on thermal performance curves.

Rationale for using a common garden approach

As is inherent to experiments carried out on samples obtained at different times of the year, one faces the decision to either carry out experiments as the samples arrive and have a confounding effect of time within the laboratory (e.g. effects of having to use different batches of media, dealing with the shelve-life of lighting systems, which may change over the course of weeks and months), or have one set of samples spend more time in the laboratory than the other. We decided for the latter, and cultured samples in a common garden prior to the beginning of the dilution experiment.

A common garden is an approach often found in ecology and evolutionary biology [3,4] (the name originating from the plant sciences): here, species or communities from different native environments are transplanted into a common environment that is different from either species' or community's native environment. If the native environment did not matter, the different organisms would rapidly display the same phenotypes in the common garden. This means that any differences we measure *despite* the time in the common garden are robust and indeed attributable to where the organisms came from. In the end, we agreed on a common garden temperature of 18ºC. From another set of experiments that measured the thermal tolerance curves (i.e. growth of the communities across a temperature gradient – currently in prep for another publication), we know that at 18ºC, for samples from spring and summer, community composition remains relatively stable, that samples can be grown to good biomass concentration in a manageable time-frame, and that growth as well as photosynthesis rates can be obtained easily. After culture in the common garden for two months (see illustrated time line below), we carried out an in-depth pilot study using lower levels of replication. Including

all trouble-shooting and analysis, this took another 5 months, during which stocks were kept in the common garden with regular tracking of growth rates and community composition, until we were confident in the methods to start the experiment as described here. While we did not find that our pilot study findings deviated from the results described here, we do not report them due to the lower replication.

It is extremely likely that our communities as they entered the laboratory, and finally, the common garden were not a perfect replicate of the communities *in situ* (especially on levels not even measured here, e.g. the bacterial and viral component), and we would ideally have kept the samples in the laboratory for much shorter time periods prior to the measurements. However, even a sample taken from the body of water and used directly on board will not be a perfect replicate (as e.g. some species might not be so amenable to the filtration process). To find a compromise between investigating near-natural communities (rather than assembling single species from culture collections) and still making use of the controllable nature of laboratory experiments, we took great effort to continuously monitor the cytometric characteristics of all samples. We provide estimates of phenotypic diversity (see below for calculation) of samples at point of freezing on the ship, and at point of entering the common garden in Figures S7 – S9. We note that this does not yield information on the identities of species present (or changes in phenotype without underlying genetic change), but does tell us how phenotypically diverse samples were throughout time, which is our main question here. We found that while some phenotypic characteristics differed between samples at t0, the initial incubator culture, and the common garden period (especially size – cells initially became a bit larger in laboratory culture), phenotypic diversity declined slightly at first, but then remained almost unchanged (see Figures S8 and S9 respectively), and were further found to not change much during the growth cycle (Figures S10 and S11)..

Below, we provide a rough time-line of the experiment (note that back-ups of stocks were kept in the common garden throughout). Numbers refer to months and start in March 2018.

Table S1: Time-line of experiment, detailing the time spent in incubation at 15ºC for spring samples (AL505), and 22ºC for summer samples (AL513), the common garden period, a pilot study, and the final experiment. Samples from the Kiel Basin grew faster than samples from the Bornholm Basin. Even though this resulted in the Kiel samples' spending more generations in the laboratory than the Bornholm samples, we can be positive that after an initial loss of species (see Figures S7-S9), time spent in the laboratory had little impact on species loss or community composition. .

	1	2	3	4	5	6	7	8	9	10	11	12	13	14	15	16	17	18	19	20	21	22
AL505																						
AL513																						
AL505 incubation 15ºC																						
AL513 incubation 22ºC																						
Common garden 18ºC																						
Pilot study at lower replication																						
Pilot study analysis																						
Full study seeded																						
Full study - growth curve 1 to µmax																						
Full study - growth curve 2 to K																						
Growth rate and composition checked at least fortnightly																						

Table S2: Sampling station overview

Below, we provide the coordinates (Long/Lat), time of sampling (spring or summer 2018 and official ALKOR identifier), as well as salinity, temperature, and nutrient content at time of sampling for each station. See also map in Figure 1 in the main text. Three technical replicates were established for each Station at each temperature and each level of dilution. StationID is as used throughout this manuscript, and not an official station identifier. The official WERUM ID is given in brackets. As each individual station was only sampled once for nutrient content, temperature, and salinity as is standard, we do not provide standard deviations as they would not carry any true meaning (technical replicates for nutrient analyses were established in the laboratory). Temperature and salinity data are as exported from the ship's CTD. Nutrient content was measured on a SEAL sequential analyser (AA3) following protocols of [12,13] upon returning to Hamburg. The March 2018 cruise, AL505, ran from 02.03.2018 to 14.03.2018. The July/August cruise 2018, AL513, ran from 29.07.2018 to 10.08.2018.

StationID (WERUM)	Time of sampling /cruise ID	Longitude	Latitude	Temperature (ºC)	Salinity	Nitrate+ nitrite (µg mL^{-1})	Phosphate (µg mL^{-1})	Silicate (µmol L^{-1})
Kiel01 (WERUM 95)	March 2018 (AL505)	11º19.36	54º31.27	1.76	11.16	53.69	18.97	12.56
Kiel02 (WERUM 05)	March 2018 (AL505)	10º08.58	54º42.42	1.28	13.15	51.58	16.29	13.12
Kiel03 (WERUM 03)	July/August 2018 (AL513)	10º20.22	54º41.7	21.35	15.00	21.17	4.56	5.44
Bornholm01 (WERUM 75)	March 2018 (AL505)	15º54.06	55º44.16	2.03	7.36	44.21	20.08	10.47
Bornholm02 (WERUM 88)	March 2018 (AL505)	15º26.02	55º16.87	2.43	7.44	46.64	22.17	12.88
Bornholm03 (WERUM 81)	July/August 2018 (AL513)	15º04.76	54º53.08	22.6	6.5	15.53	2.22	4.87

Setting up the dilution experiment

To set up the dilution experiment, we first counted cell numbers in the Kiel Bight community samples (3 stations, liquid culture, non-frozen samples) and Bornholm Basin community samples (3 stations, liquid culture, non-frozen samples) using a BD Accuri C6 flow cytometer. More than 3 stations per basin had been obtained on board, but for the sake of keeping the total number of experimental units within a manageable range, we focused on 3 stations per basin. The cell counts also yield flow cytometric fingerprints that allow for an estimate of phenotypic diversity or trait-level diversity [5] which is largely based on photo-pigment composition and size [6] [7] (see below for more details). Samples were then diluted in six 10-fold dilution steps at the appropriate salinity, down to the lowest point of dilution (in theory containing no more than 1 species or pico-phytoplankton per mL). Six technical replicates of each sample in the dilution series (i.e. region*station*dilution) were left to regrow to 10^6 cells mL^{-1} at the assay temperatures of 15ºC, 18ºC, and 22ºC. These temperatures are all within the ranges of temperatures commonly experienced during late spring (15ºC), summer (18ºC), and the height of summer (22ºC). This resulted in a total of 648 unique experimental units. From the time of dilution, samples were cultured on 48 well plates (1.5mL), which provide a space- (and plastic) saving alternative to larger culturing vessels. We had tested beforehand that between-treatment differences did not change significantly with the culture vessels used.

Then, we re-diluted all samples to 3000 cells mL^{-1} and tracked two consecutive growth curves: One, where samples were harvested for net photosynthesis measurements at µmax, followed by a full growth curve to carrying capacity (ca. 23 days, see below for details as not all samples reached K the same day) in all experimental units at all temperatures, with measurements taken on the flow cytometer every other day. We found that growth at µmax did not differ between the first and second growth cycle. Yet, growth hinged on a

combination of dilution, region of origin, and assay temperature. As a result, the points of µmax and carrying capacity were not reached on the same day for all samples in either growth cycle. Supporting Figure S17 has the growth rates at µmax, and Table "20200606_timetoK.csv" on data dryad details the times at which carrying capacity was reached. We would like to point out that in order to keep the experiment manageable, different growth rates indeed are an advantage rather than a disadvantage, as measurements can be spread out throughout multiple days and importantly can be carried out at the same time of day for each sample to account for effects of circadian rhythms on metabolic processes.

Estimation of cell size

Cell size as diameter in µm was obtained from the flow cytometer's forward scatter after calibration with size beads. Taking into account cell counts per mL and assuming on average spherical shapes and using conversion factors after [8], we then calculated an estimate of pg carbon per mL to obtain biomass produced.

Estimation of Net Photosynthesis

Net photosynthesis rates were obtained when samples were in exponential phase, on PreSens ® SDR Sensor Dish optodes. Here, we aimed for a total of 10^4 - 10^5 cells in 4mL measurement vials (the optodes sit on the bottom of each vial). To achieve this cell density, aliquots from the harvested experimental units had to be diluted in the appropriate media and salinity. PreSens optodes are pre-calibrated by the manufacturer. A headspace of oxygen can be eliminated by filling samples to the rim and sealing off with parafilm. We measured oxygen production for 15 minutes in the light, and respiration for 15 minutes in the dark. Whenever an experimental unit was run on the PreSens optode, it was also run (in its diluted state) on the flow cytometer to allow for per cell estimates. Net photosynthesis was calculated

considering that phytoplankton in our set-up will only be able to photosynthesise during the light phase (12 hours), but will respire throughout the day *and* night phase (24 hours). All measurements were carried out at the same time of day (~ 9am to 11am) under the light- and temperature conditions set in the incubator (i.e. all experimental units at their assay temperatures).

Molecular analysis of diversity (as species richness)

We obtained two measures of biodiversity in our samples. One, following CTAB DNA extractions [9], a subset of representative samples was sent for DNA- meta-barcoding at biome-id (16S primers: forward CCTACGGGNGGCWGCAG, and reverse GACTACHVGGGTATCTAATCC, 18S primers: forward CCGCGGTAATTCCAGCTC and reverse CCTTGGTCCGTGTTTCTAGAC), resulting in a MOTU (meta-barcoding operational taxonomic units) estimate for those samples. A MOTU is grouped by DNA sequence similarity of a specific taxonomic marker gene, here 16S and 18S.

In total, we sent off three DNA pellets for each region for each dilution step. As we found that MOTU scales well with phenotypic diversity, we forewent further MOTU analyses in favour of cheaper and faster phenotypic diversity measurements.

Flow cytometric analysis of diversity

As molecular analyses are infamously costly, we chose phenotypic diversity [7] as our second measure of diversity. This was assessed using the parameters returned by the flow cytometer (abbreviations in Table S4).

On slow sampling rates of 14μL/minute, we used an aliquot of 50μL of each unique experimental unit to obtain detailed cytograms (for tracing growth curves, 10-20μL often suffice and flow rates can be chosen at faster settings). Larger aliquots do not yield better

cytograms, and only serve to clog up the flow cytometer. The aliquot taken from each experimental unit was replaced by nutrient-free medium of the correct salinity and the resulting (small) dilution was incorporated in the growth rate measurements.

We first stained aliquots of the sample with SYBR Gold, alongside a 0.2µm filtered MiliQ sample. This allows us to distinguish debris and cytometer noise from living matter (see below) on the FL1 channel (FITC in cytogram display). Below, we show an example for a thresholded fraction containing debris, as well as bacterial (E4), viral (R3), and assumedly pico-eukaryotic matter (E3 along –side beads of known size (R2, 1µm microspheres from invitrogen), with FITC on the y and SSC on the x axis. Our gating strategies are in line with [10].

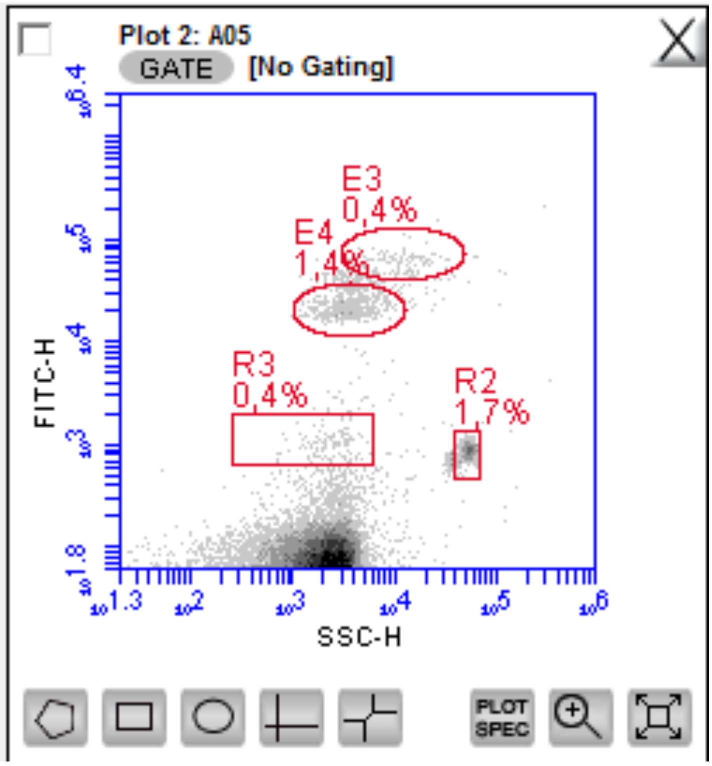

Figure S1: Thresholded fraction containing debris, as well as bacterial (E4), viral (R3), and assumedly pico-eukaryotic matter (E3 along –side beads of known size (R2, 1µm microspheres from invitrogen), with FITC on the y and SSC on the x axis. See also our data dryad files on https://doi.org/10.5061/dryad.0p2ngf1xw.

Knowing where the DNA positive clouds lie, and which parts to exclude as debris/cytometer noise, we then further gated for FL3 (Chl-a proxy) positive organisms (DNA positive but FL3 negative were also tracked to get an idea of the heterotrophic fraction, but not used for this study) comparing known bacteria, known single species phytoplankton, and community samples. Depending on whether one is interested in tracking the bacterial compound, one either choses to only count organisms within the FL3 positive gate or quadrant, or alternatively, one can set the thresholds so that very small, low-FL3 events are automatically excluded from the display.

Below, we provide an example of FL3 (PerCP) against FSC with a fairly high threshold of 2500 on FSC and FL3. This is the fingerprint of a fairly high diversity Baltic Sea community sample. We would consider events in Q1-UR for further analysis.

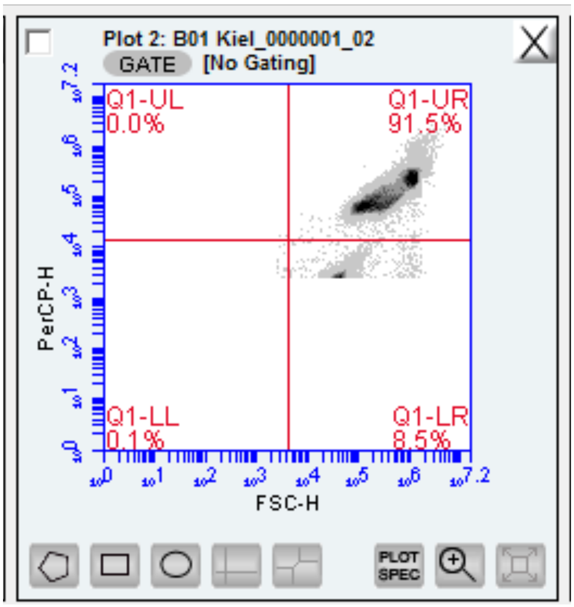

Figure S2: An example of FL3 (PerCP) against FSC with a fairly high threshold of 2500 on FSC and FL3. This is the fingerprint of a fairly high diversity Baltic Sea community sample. We would consider events in Q1-UR for further analysis. See also our data dryad files on https://doi.org/10.5061/dryad.0p2ngf1xw.

An example of FL3 (PerCP) against FSC with a much lower threshold (250 on both) is available on data dryad (https://doi.org/10.5061/dryad.0p2ngf1xw). Note that the location of

the picoplankton cloud on the cytogram may differ throughout the cytograms presented here, resulting from a breakdown of our flow cytometer half-way through the experiment, which necessitated repeated re-calibrations (e.g. of the rental flow cytometer, and the original flow cytometer after repair) associated with a shift in the ‘absolute’ location of the cloud.

Importantly, the chosen gates, quadrants, or thresholds, still allow us to pick up organisms that might not have their main photosynthetic pigments detected by FL3, as even organisms higher in FL2 and FL4, but low in FL3 will fall within this gate, but not the debris. An example can be found below for one of our high and low diversity experimental units:

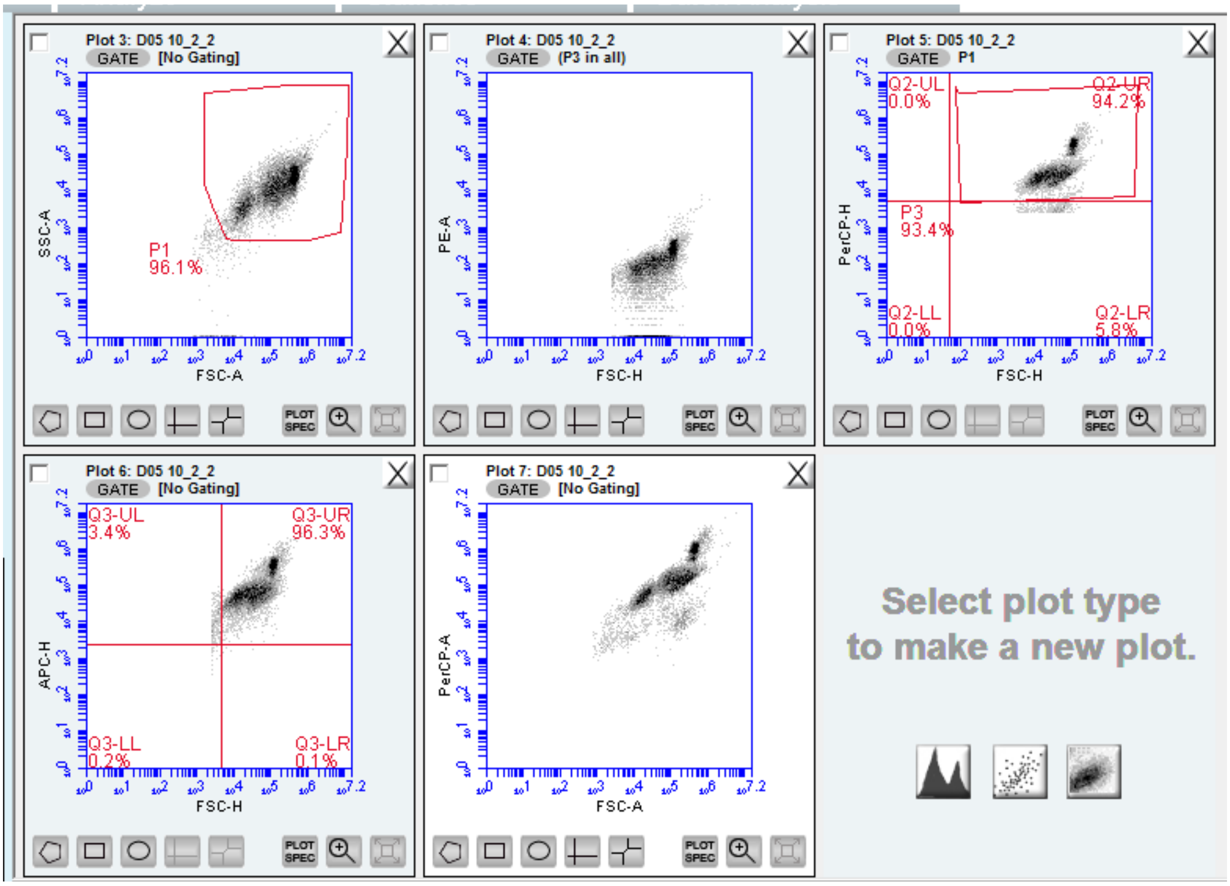

Figure S3: An example cytogram for SSC, FSC, FL2, FL3, and FL4 in one of the high diversity experimental units.
See also our data dryad files on https://doi.org/10.5061/dryad.0p2ngf1xw

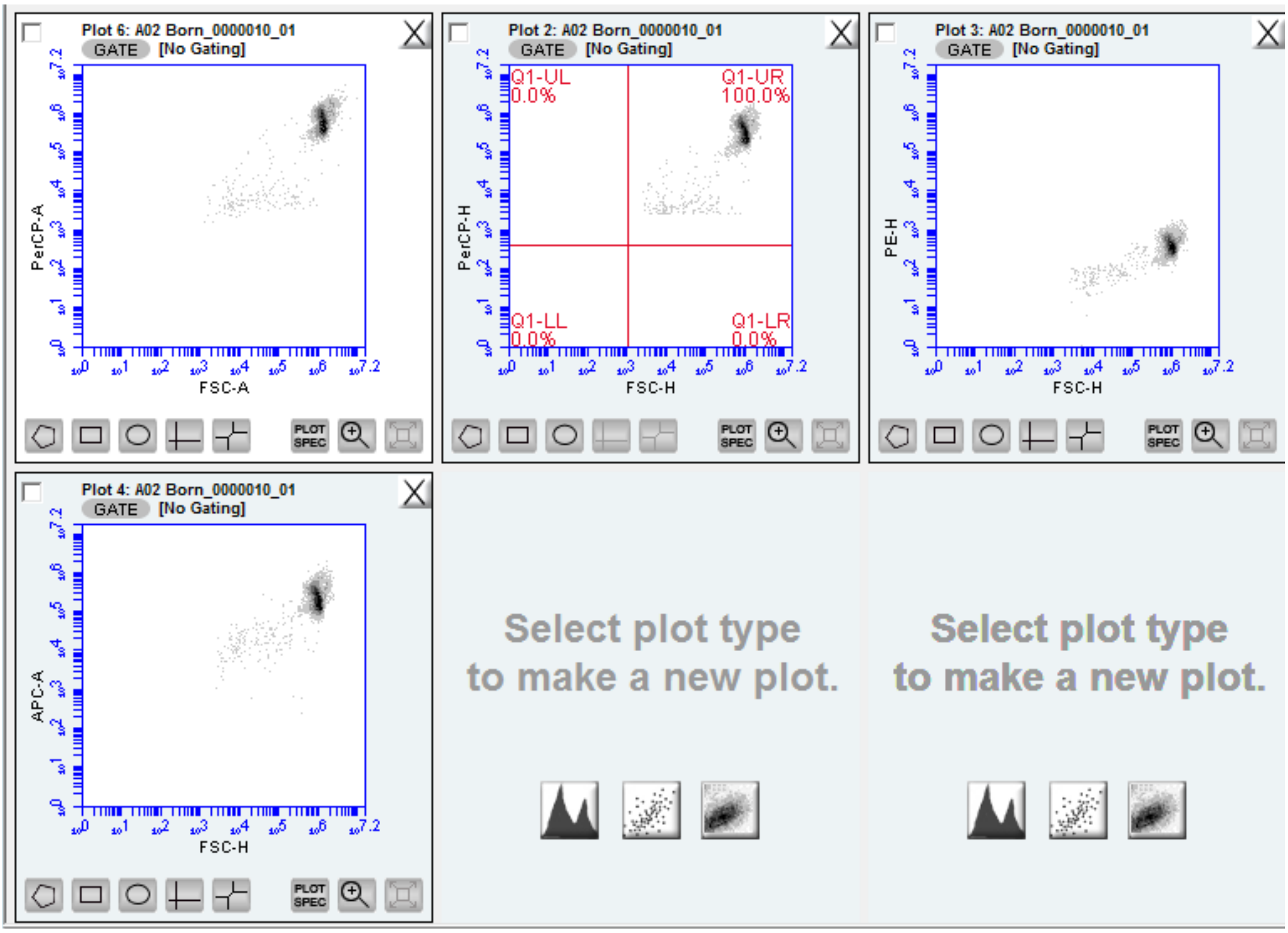

Figure S4: An example cytogram for SSC, FSC, FL2, FL3, and FL4 in one of the low diversity experimental units. See also our data dryad files on https://doi.org/10.5061/dryad.0p2ngf1xw

We also compared the chosen gating region to beads of known size and a known *Ostreococcus* (a picoplankton of about 1.5µm diameter) sample, to make sure we were capturing the full picoplankton community. Beads of known size run without an organism will result in cytograms akin to this (beads close to the size of the fraction under examination can also be run alongside the sample for direct comparison. On data dryad (https://doi.org/10.5061/dryad.0p2ngf1xw), we show the range of calibration beads before and after the cytometer was sent for repairs (here, data have been exported from the Accuri and visualised in R using the FlowCore package version 2.0 and ggplot2 version 3.3.1.), as well as a cytogramm for a known and fairly clean *Ostreococccus* sample.

For each individual organism of the population, this approach yields the raw flow cytometry data with an individual measure for size (FSC), granularity (SSC), as well as FL2, FL3, and FL4 (depending on brand/version number of flow cytometer these are PE, PerCP and APC). The resulting matrices can then be used within the PhenoFlow package to calculate within-sample diversity (akin to alpha diversity) by first trialling the 'bin width' using the FlowBase() function within the package (i.e. we have to iterate through a number of 'bins' that tell us when two cells or sets of parameters are significantly different. This step is time-consuming but necessary). This yields a frequency distribution of cells with a certain attribute (or set of attributes). Based on the frequency dataframe, we can calculate alpha diversity using the Diversity() function (which utilises a bootstrapping approach). The resulting values are not very meaningful *per se*, but once we have a per-sample estimate, we can track how phenotypic diversity changes through time, or differs between samples. A step-by-step guide on how to use the package can be found on the following github: https://github.com/rprops/Phenoflow_package/wiki/1.-Phenotypic-diversity-analysis

The matrices containing the raw flow cytometry data can also be used to compare samples to each other (also throughout time), akin to beta diversity, *via* any code that creates similarity or dissimilarity matrices, i.e. a simple PCA or NMDS plot for graphic representation or a PERMANOVA for statistical analysis (we use the R package vegan for this purpose). We find that for comparing how samples change through time or how samples from different regions differ from each other, a similarity/dissimilarity matrix based on means rather than individual measures, yields the same results as calculations based on individual cell measurements, but at much faster computing speeds (a few minutes compared to more than an hour). We make a point that where time or computer power is a limiting factor, using mean data frames is a valid option.

We would like to add that for this manuscript, the gating on the Accuri software is merely to aid the researcher as they observe the samples being counted (e.g. to immediately spot contaminants or issues with the cytometer). We exported the full raw fcs data files for gating and de-noising to be carried out in R (following the same gating steps) within the PhenFlow() package. We provide higher quality versions of the cytograms shown here on datadryad (https://doi.org/10.5061/dryad.0p2ngf1xw). The authors are happy to provide raw fcs files upon reasonable request.

Statistical analysis

All data were analysed in the R programming environment (version 3.5.3.). To analyse the shape of the growth curves, non-linear curve fitting of a baranyi growth model [11] was carried out using the 'nlsLM' function in the R package, 'minpack.lm'(version 1.2-1). Parameter estimation was achieved by running 1000 different random combinations of starting parameters for cell count at carrying capacity, duration of lag phase, and maximum growth rate picked from a uniform distribution. The script then retains the parameter set that returned the lowest Akaike information criterion (AICc) score. Parameters (biomass and cell size at carrying capacity, net photosynthesis during exponential growth) were then compared through a mixed effects model (within the nlme package, version 3.1-137). There, the respective parameters were explained by a global model that included sampling location (Kiel Bight or Bornholm Basin), assay temperature (15ºC,18ºC, or 22ºC), and dilution step (from lowest to highest) and sampling season (spring or summer) as fixed factors in full interaction. Sampling station was computed as a nested random effect within region. In all cases, seasonality was found to not explain the data better and was subsequently dropped from the fixed factors to avoid over-parameterisation of the model. For multi-model selection, we computed small sample-size corrected AIC scores (AICc) and then compared the models by

calculating delta AICc values and AICc weights using the “MuMIn” package (version 1.42-1). We picked the model where delta AICc was > 2 for refitting with REML. PERMANOVAs were carried out using the “ecodist” (2.01) packages. Distance matrices using the Bray–Curtis index were created from these, on which we ran PERMANOVAs to test for separation of samples by treatment. Pairwise contrasts between treatments were examined *via* the function permdisp() followed by TukeyHSD post-hoc tests.

For graphical presentation of data, we used the ggplot2 (version 3.2) and vegan (version2.4) packages. While NMDS plots are common for the comparison of ecological sampling sites, we found that the distance matrices did not differ significantly from PCA plots, and are presenting the latter throughout for their more direct compatibility with PERMANOVA results.

Further Supporting Figures

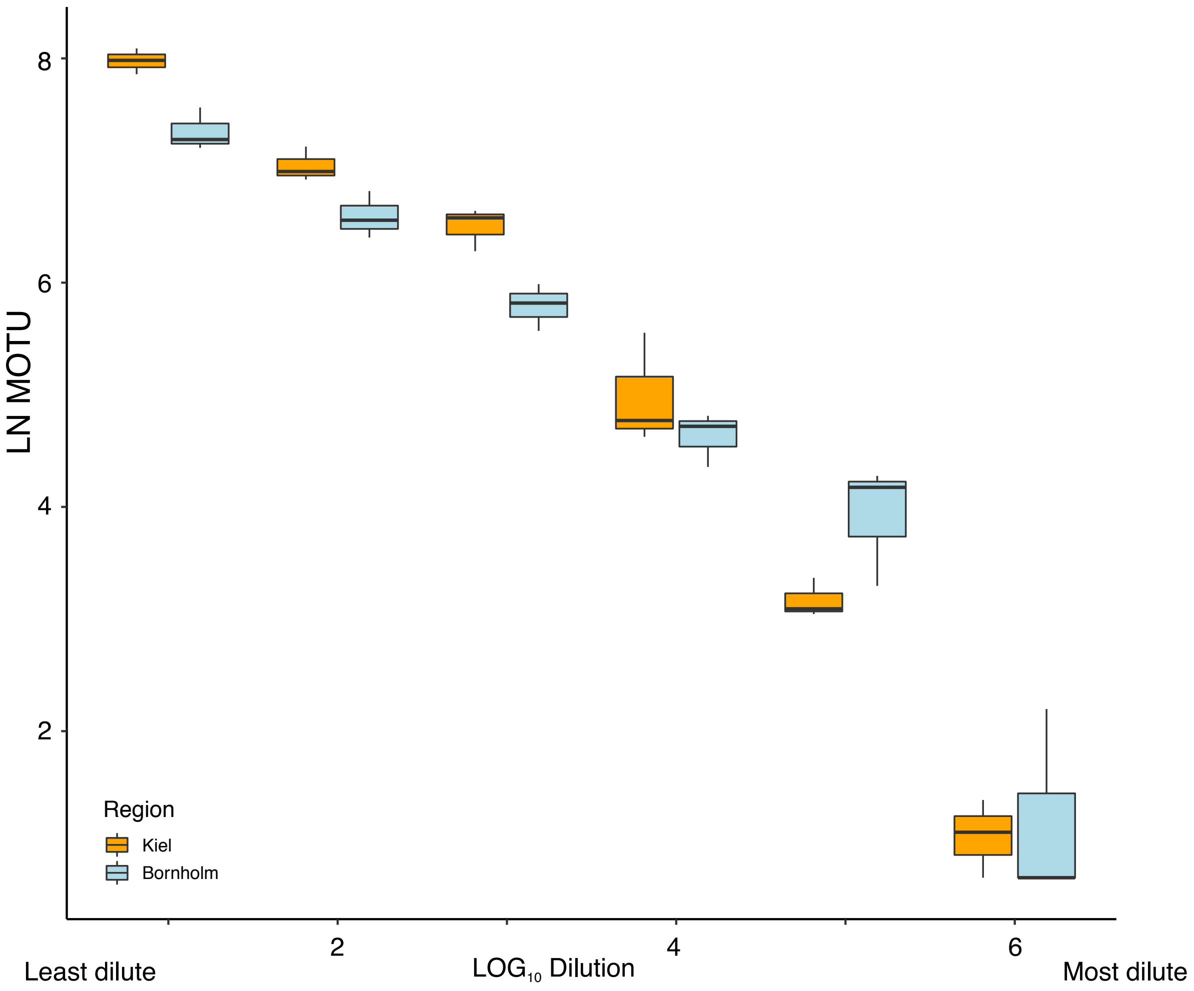

Figure S5: Dilution is strongly correlated to MOTU (operational taxonomic units obtained through meta barcoding)

The relationship between the logarithms of dilution and MOTU count reveals that the dilutions successfully reduced species richness in Kiel Bight (orange) and Bornholm Basin (blue) samples. Kiel samples had slightly higher original MOTU counts, which was driven largely by a slightly lower species count and higher predominance of cyanobacteria in the Bornholm Basin samples during the summer (see also below). The boxplots are displayed as is standard, with the girdle band indicating the median, and the whiskers extending to the 25th and 75th percentile. For each unique treatment combination (dilution*region*temperature). Due to the high costs involved in meta barcoding we sent off 3 samples for each region and dilution (after common garden culture at 18ºC). (This is a larger version of subpanel C in Figure 1 in the main manuscript).

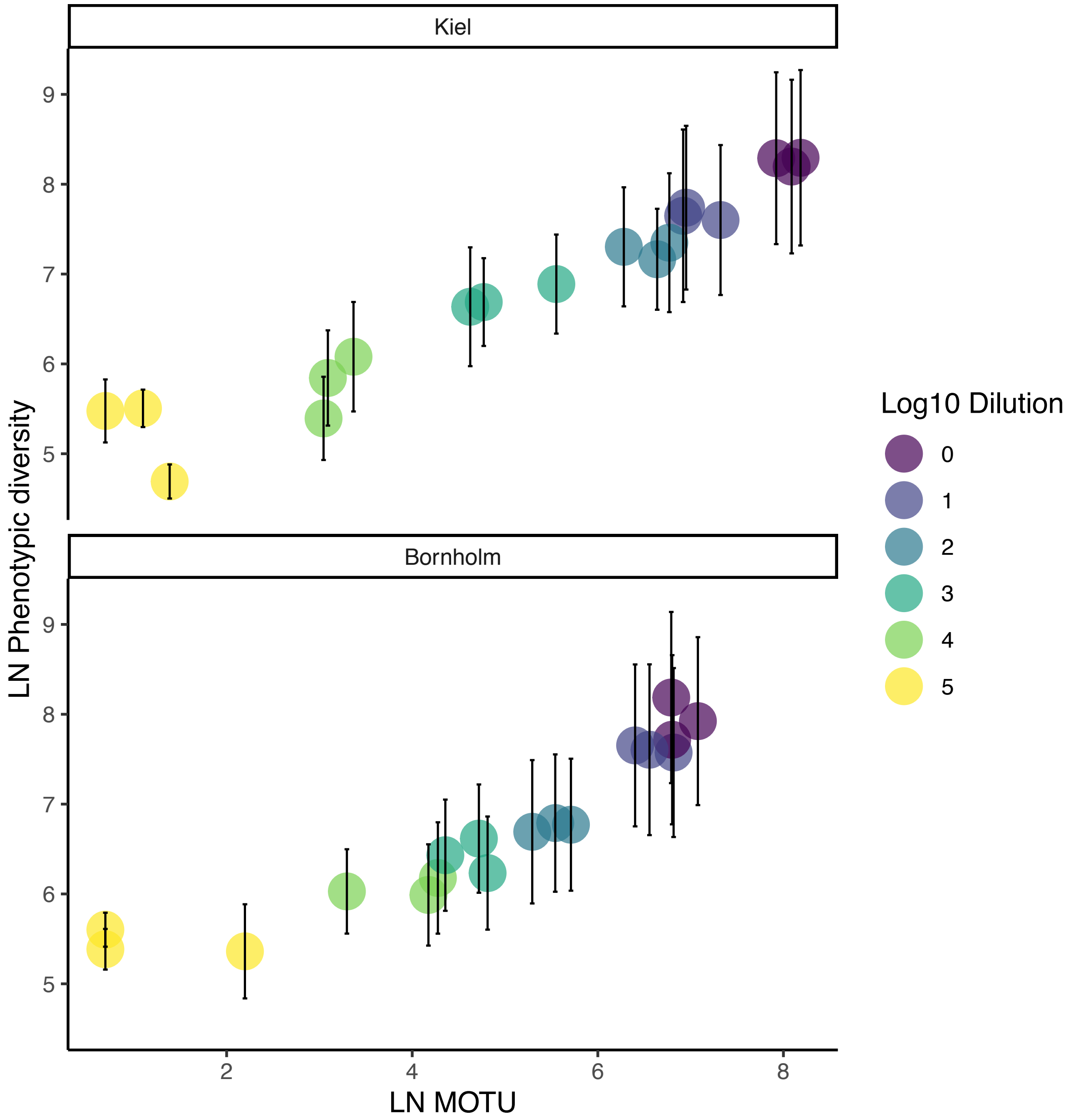

Figure S6: Phenotypic diversity scales with MOTU diversity
For phytoplankton communities from the Kiel Bight (upper panel) and the Bornholm Basin (lower panel) phenotypic diversity scales well with the dilution steps (yellow for most dilute i.e. lowest species richness, purple for least dilute, i.e. highest species richness) and the species richness returned through meta-barcoding (MOTU). For each dilution, we display means across six biological replicates within three stations. The errorbars are for ±1SD for phenotypic diversity.

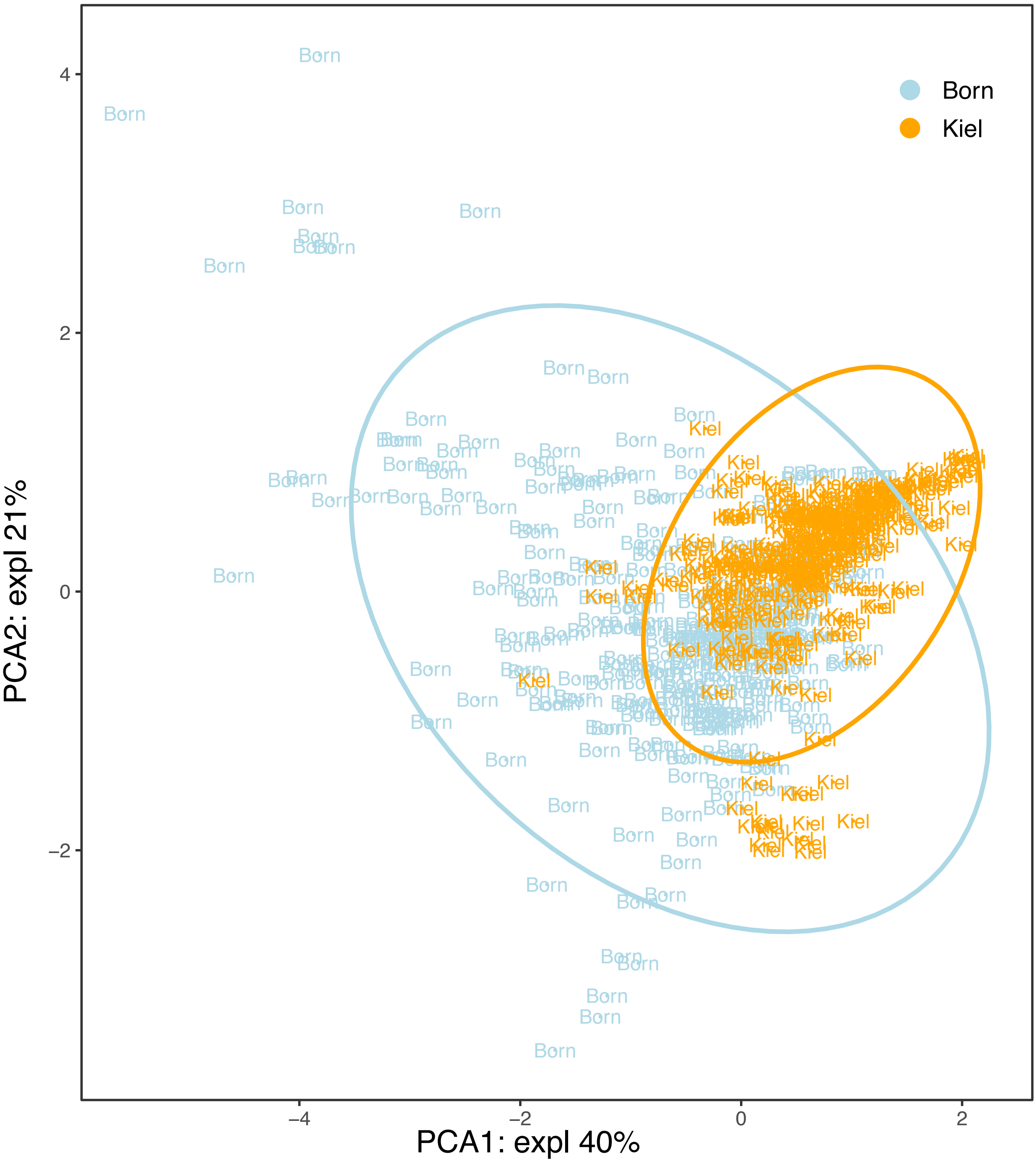

Figure S7: Phenotypic characteristics for Kiel Bight and Bornholm Basin samples did not differ significantly after time in the common garden

We investigated the phenotypic characteristics of all experimental units, i.e. cell size (*via* FSC), granularity (*via* SSC), and photosynthetic pigments (obtained *via* the FL2, FL3 and FL4 channels). PERMANOVAs showed, that samples did not differ significantly in their phenotypic composition ($F_{1,13}$= 2.72, p =0.058) and a cross-check with the species identities returned from meta-barcoding showed that any differences were driven solely by the summer months seeing a higher abundance of cyanobacteria in the Bornholm region.

Here, each "Kiel" or "Bornholm" identity on the plot contains the mean information on phenotypic characteristics per unique experimental unit (a plot with this information per cell per sample would be beyond readable). (This is a larger version of subpanel C in Figure 1 in the main manuscript). In Figures S8 and S9 we show that while this phenotypic composition differed slightly to samples at t0 (i.e. frozen directly after filtration), phenotypic composition then remained largely stable between the time spent in the incubators at 15º/22ºC and the

common garden at 18ºC. Further, phenotypic diversity also remained largely unchanged, e.g. while cells *on average* increased in size after being brought to the laboratory, diversity eventually stabilised.

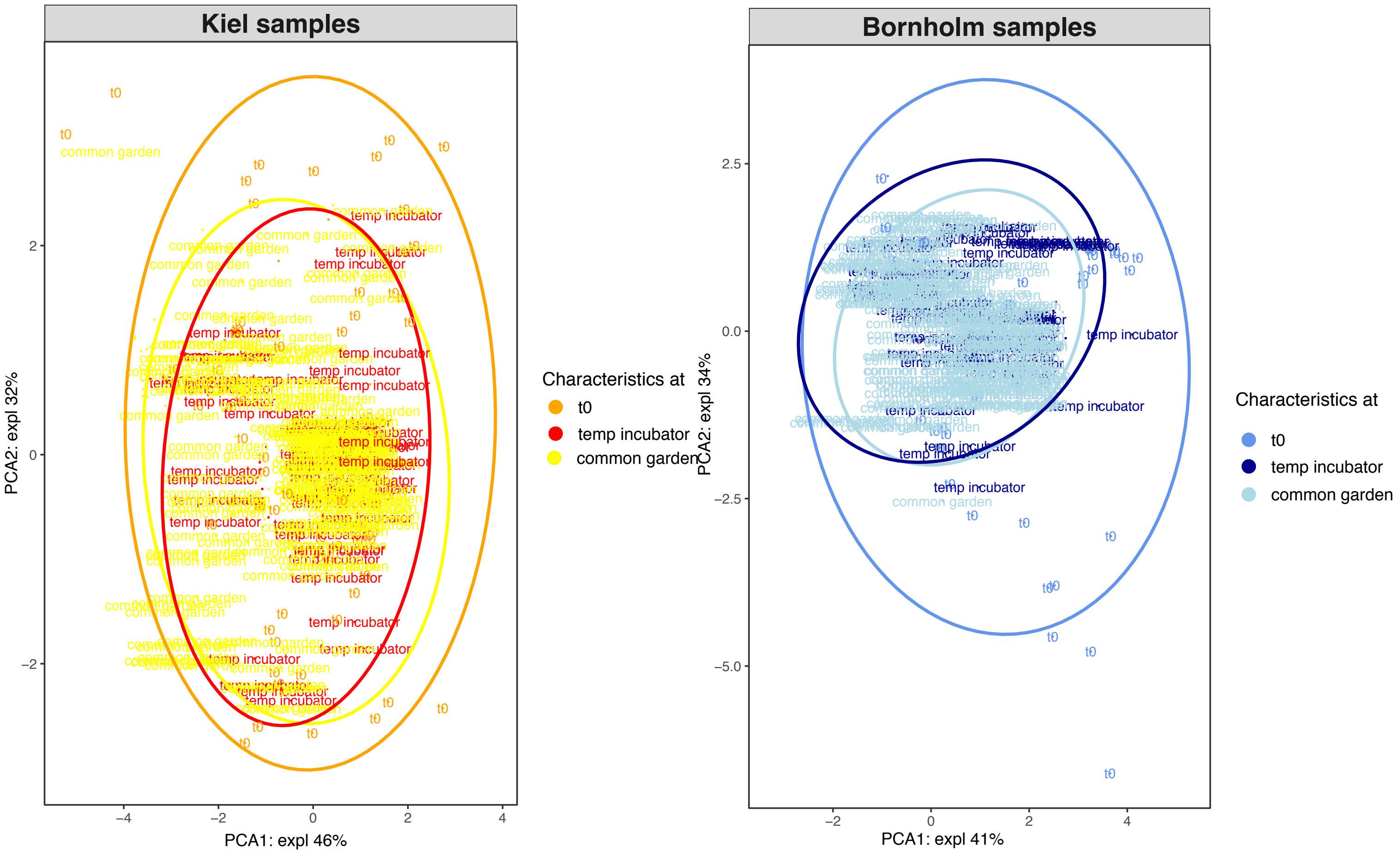

Figure S8: Phenotypic characteristics for Kiel Bight and Bornholm Basin remained largely stable between incubation at 15ºC or 22ºC and the common garden period, but differed slightly from t0

Phenotypic characteristics as detailed in the methods and also shown in Figure S2 remained stable between culture in the incubators set to 15ºC for March 2018 samples/22ºC for July/August 2018 samples and the common garden at 18ºC. As is to be expected, there were some differences to the original samples ("t0") frozen in sorbitol immediately after on-board filtration. Phenotypic diversity decreased slightly during laboratory culturing,

but eventually stabilised (see Figure S9). Each “t0” or “temp incubator” or “common garden” identity on the plot contains the *mean* information on phenotypic characteristics per unique experimental unit, with several measurements carried out for each time point per station per replicate.

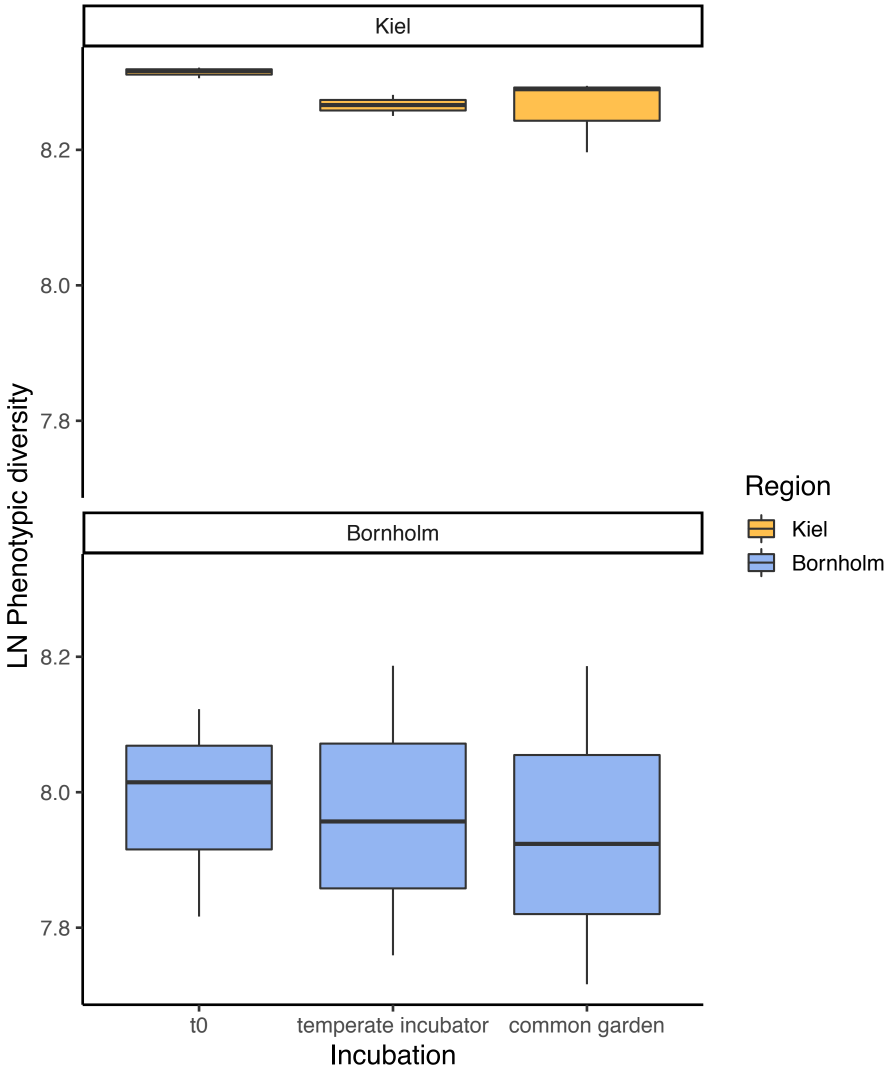

Figure S9: Phenotypic diversity for Kiel Bight and Bornholm at t0, incubation at 15ºC or 22ºC and the common garden period at 18ºC

Phenotypic diversity calculated from the flow cytometric characteristics was overall higher in the Kiel Bight (orange) phytoplankton communities than for those from the Bornholm Basin (blue). There was an overall (but slight) decline in phenotypic diversity as time proceeded, and this was the most pronounced early on (t0 to incubator). For each individual boxplot, we have pooled the data of the samples from all stations. The boxplots are displayed as is standard, with the girdle band indicating the median, and the whiskers extending to the 25th and 75th percentile.

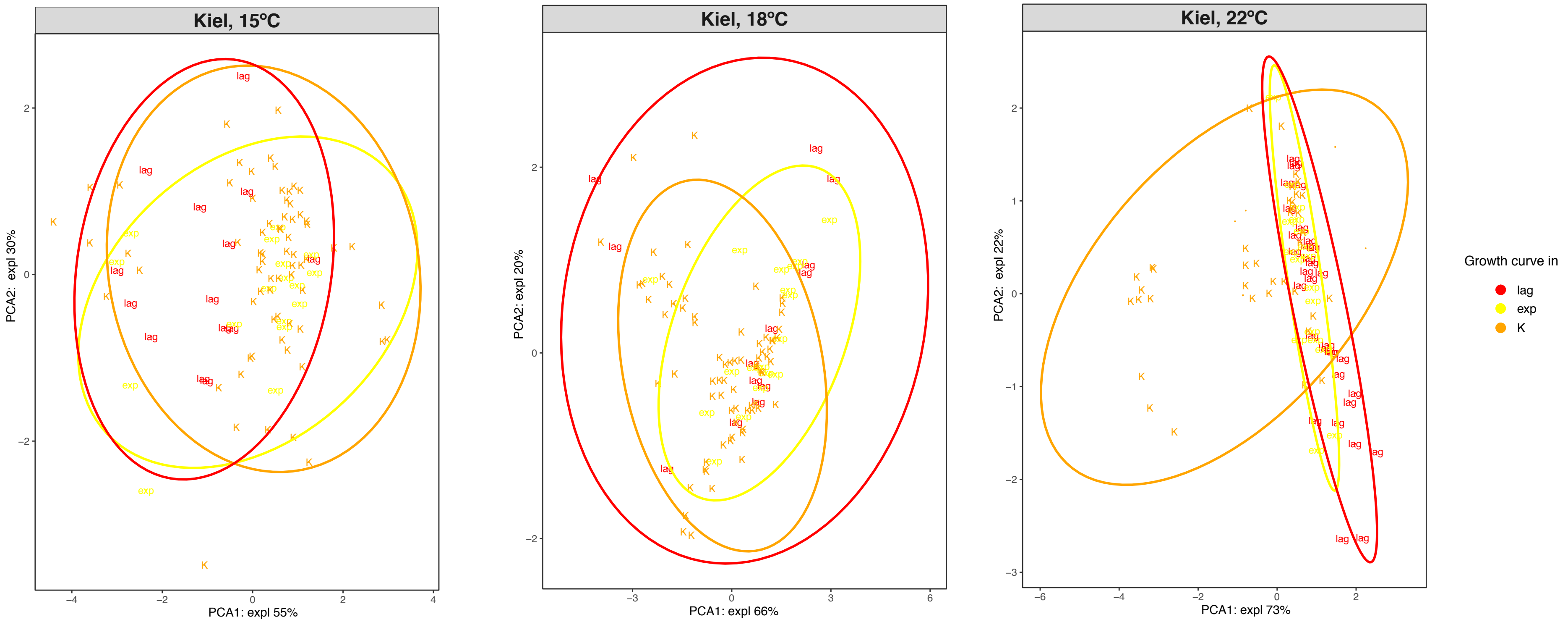
Kiel, 15ºC
Kiel, 18ºC
Kiel, 22ºC
PCA2: expl 30%
PCA1: expl 55%
PCA2: expl 20%
PCA1: expl 66%
PCA2: expl 22%
PCA1: expl 73%
Growth curve in
lag
exp
K

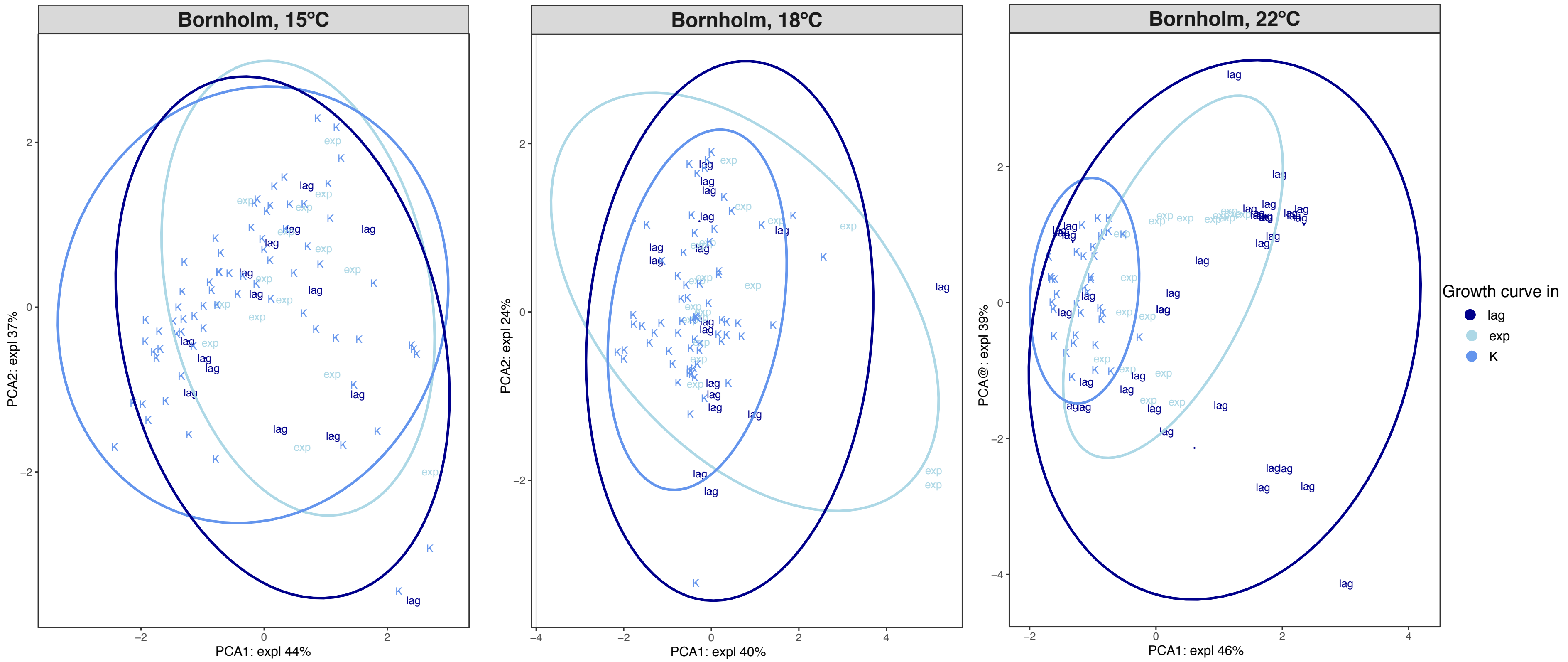

Figure S10: Once established through dilution, community phenotypic characteristics remained largely stable throughout the growth cycle. In the cytometric output for communities from the Kiel Bight (upper row, red tones) and the Bornholm Basin (lower row, blue tones), we can see that there was no overall significant change in community characteristics throughout the growth curve (PERMANOVA $F_{2,13}$= 2.35, p =0.08). However, at 22ºC Kiel Bight communities at carrying capacity seemed to develop a lower chlorophyll phenotype, and during the lag phase, Bornholm Basin communities at 22ºC showed on average higher cell size. We show in Figure S11 that this did not affect phenotypic *diversity* significantly throughout the growth cycle. Lag is for lag phase, exp for exponential phase, and K for carrying capacity

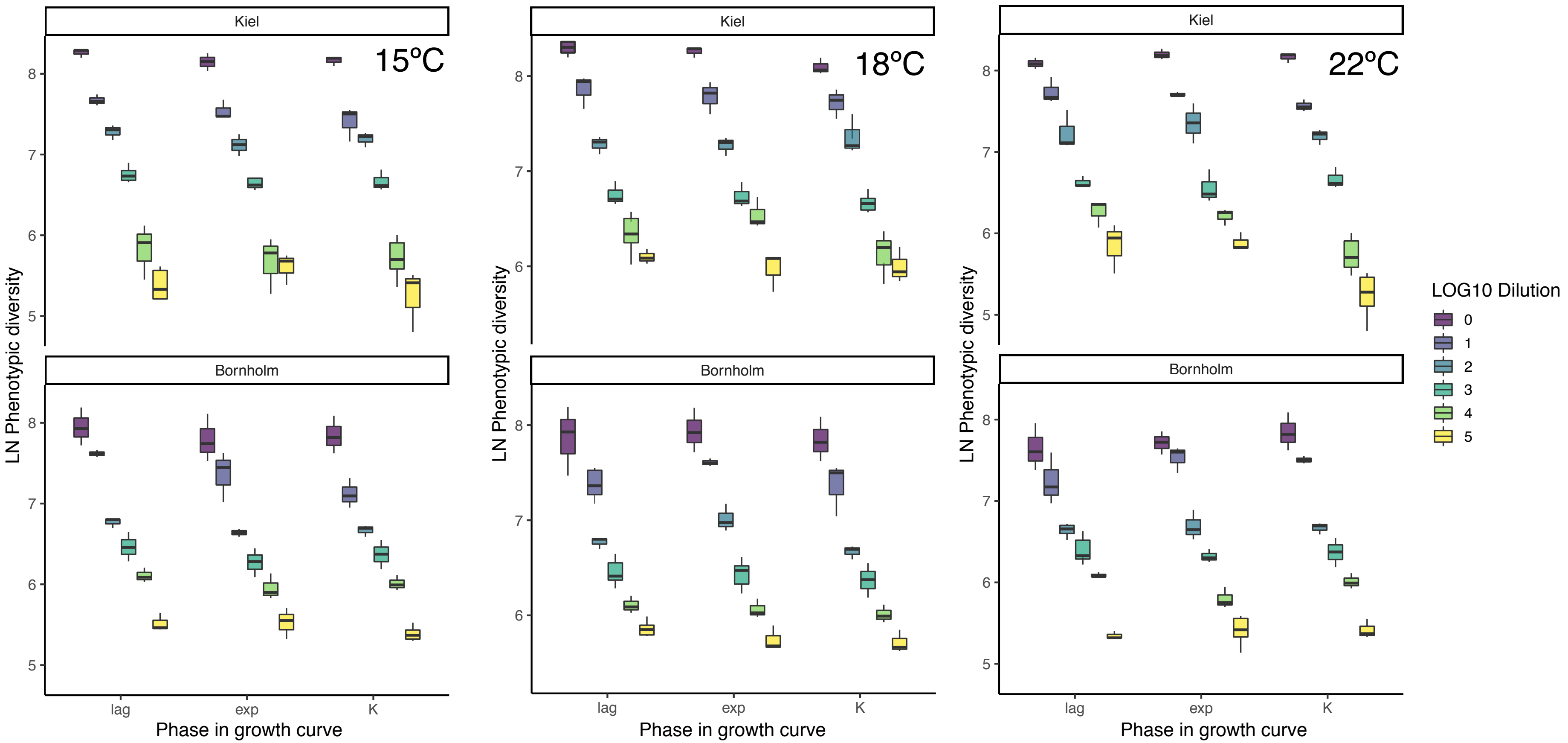

Figure S11: Once established through dilution, community phenotypic diversity also remained largely stable throughout the growth cycle. In communities from the Kiel Bight (upper panel) and the Bornholm Basin (lower panel), phenotypic diversity was not significantly affected by the phase of the growth (lag for lag phase, exp for exponential phase, and K for carrying capacity) across dilutions (purple = least dilute i.e. highest species richness, yellow = most dilute, i.e. lowest species richness). For each individual boxplot, we have pooled all six replicates for all three stations. The boxplots are displayed as is standard, with the girdle band indicating the median, and the whiskers extending to the 25th and 75th percentile.

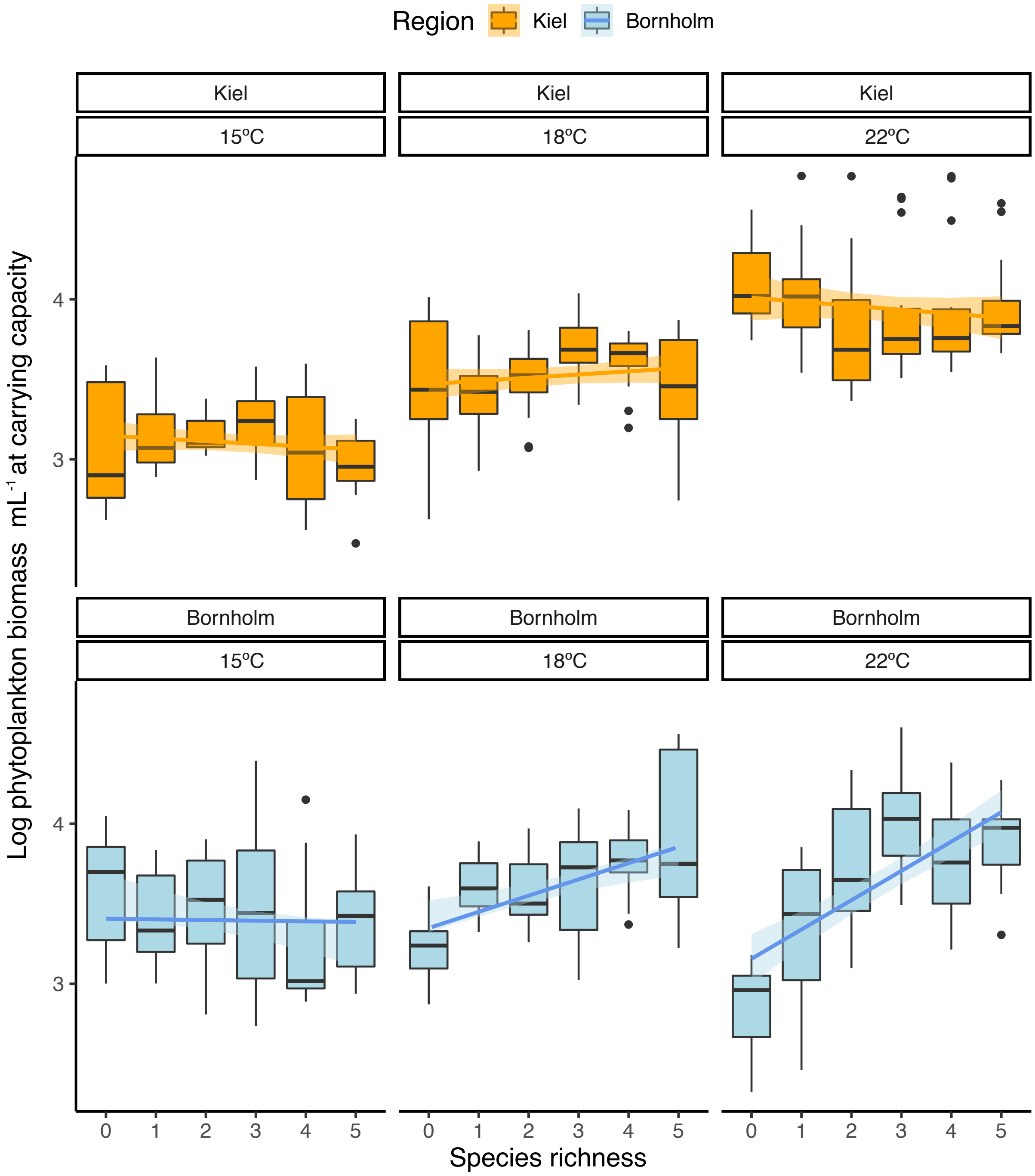

Figure S12: Biomass at carrying capacity K

Biomass at carrying capacity (here in pg C per mL, displayed as LOG10 for clarity) in samples from the Kiel sampling stations (orange, upper) and the Bornholm sampling stations (blue, lower) was influenced by assay temperature (individual panels) and dilution (labelled as 'species richness'. Here, displayed as the LOG10 of phytoplankton cells after dilution, which is a good indicator for species richness (see Figure S5 and main manuscript Figure 1). A slope that does not deviate significantly from 0 (see also Table S5) indicates that functional redundancy is high. A slope that does deviate significantly from 0 indicates that species richness has a strong impact on the trait under investigation, with positive slopes for samples with low functional redundancy. While temperature has an impact on biomass at carrying capacity rates in the samples from the Kiel Area (highest rates at 18ºC, lowest at 15ºC, and intermediate values for 22ºC), there is no significant impact of loss of rare species (i.e.

dilution). The boxplots are displayed as is standard, with the girdle band indicating the median, and the whiskers extending to the 25th and 75th percentile. For each unique treatment combination (dilution*region*temperature), n=6. Standard deviations for the slopes can also be found in Table S5. Shaded areas in the plot are confidence intervals generated in R and mainly for graphical representation.

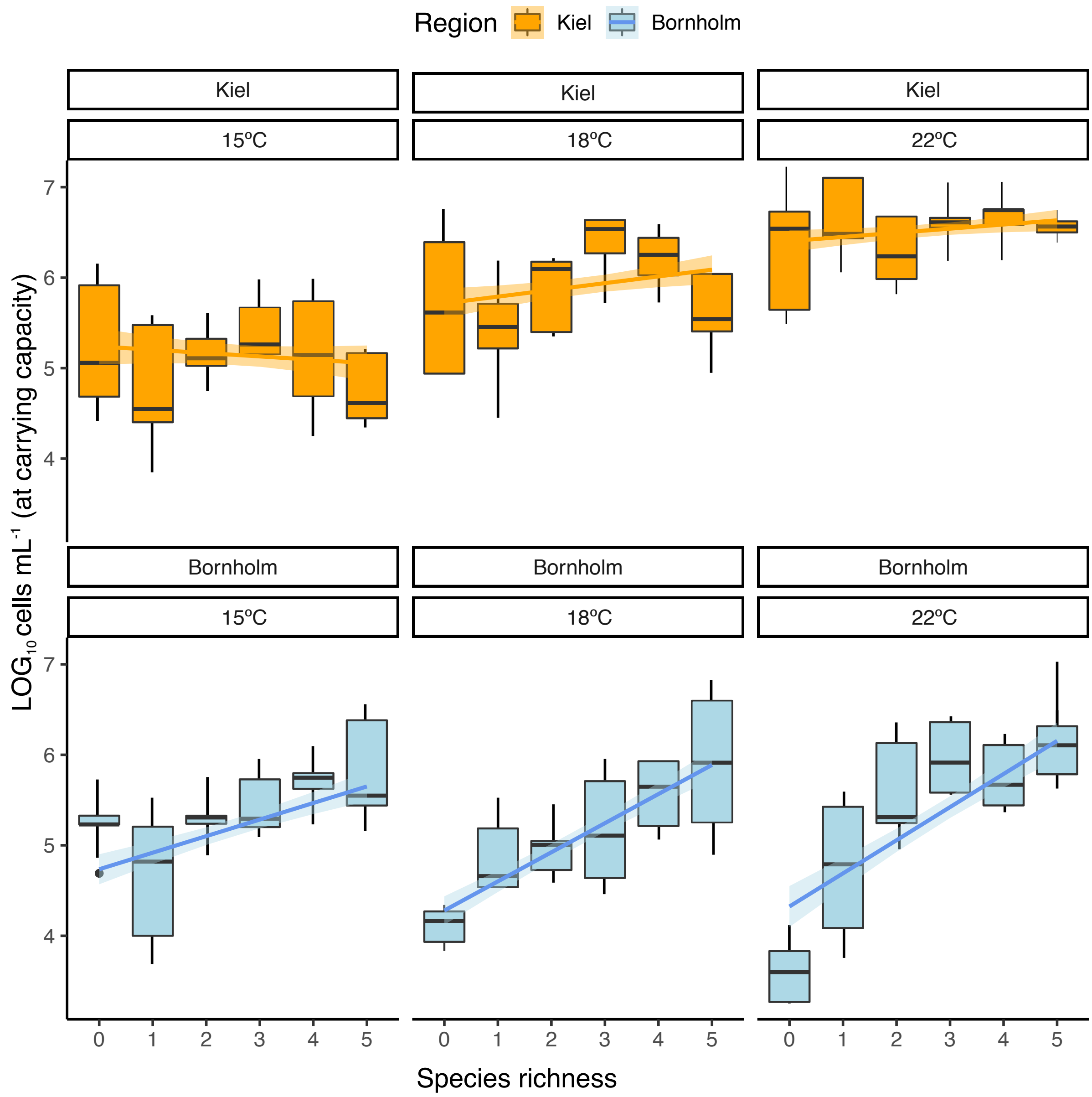

Figure S13: Cell count at carrying capacity

Cell count mL^{-1} at carrying capacity (here displayed as LOG10 for clarity) in samples from the Kiel sampling stations (orange, upper) and the Bornholm sampling stations (blue, lower) was influenced by assay temperature (individual panels) and dilution (labelled as ‘species richness’. Here, displayed as the LOG10 of phytoplankton cells after dilution, which is a good indicator for species richness (see Figure S5 and main manuscript Figure 1). A slope that does not deviate significantly from 0 (see also Table S5) indicates that functional redundancy is high. A slope that does deviate significantly from 0 indicates that species richness has a strong impact on the trait under investigation, with positive slopes for samples with low functional redundancy. While temperature has an impact on cell count at carrying capacity rates in the samples from the Kiel Area (highest rates at 18ºC, lowest at 15ºC, and intermediate values for 22ºC), there is no significant impact of loss of rare species (i.e. dilution). The boxplots are displayed as is standard, with the girdle band indicating the median, and the whiskers extending to the 25th and 75th percentile. For each unique treatment combination (dilution*region*temperature), n=6. Standard deviations for the slopes are in Table S5. Shaded areas in the plot are confidence intervals generated in R and mainly for graphical representation.

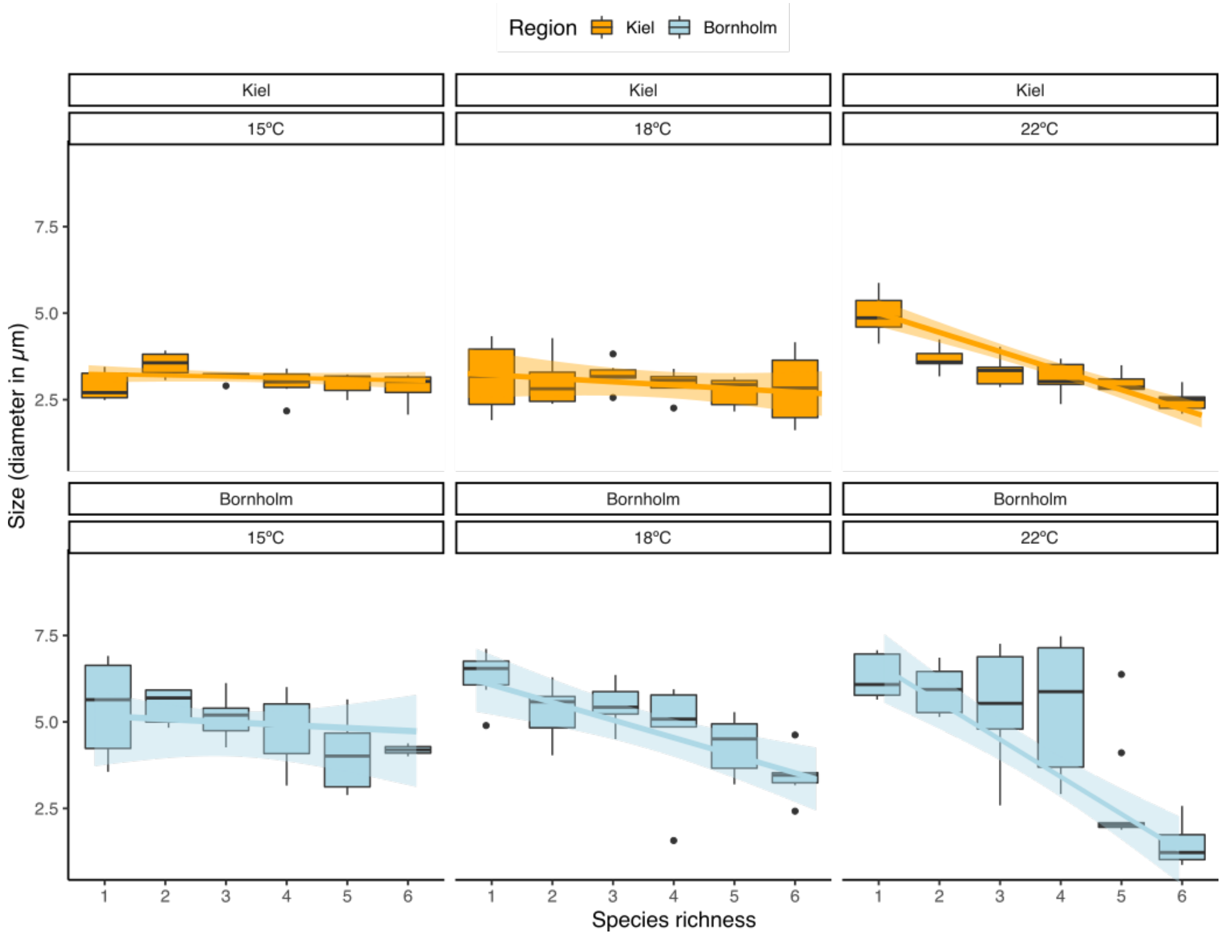

Figure S14: Size (diameter in µm) at carrying capacity as used for biomass estimation

At carrying capacity, cell diameter in samples from the Kiel sampling stations (orange, upper) and the Bornholm sampling stations (blue, lower) was influenced by assay temperature (individual panels) and dilution (here, displayed as the LOG10 of phytoplankton cells after dilution, which is a good indicator for species richness (see Figure S5 and main manuscript Figure 1). At 15ºC, dilution did not significantly affect cell size. At 18ºC, dilution affected cell size only in samples from the Bornholm region. At the highest temperature (22ºC), cell size strongly decreased when communities were more diverse in samples from both regions. A slope that does not deviate significantly from 0 (see also Table S5) indicates that functional redundancy is high. A slope that does deviate significantly from 0 indicates that species richness has a strong impact on the trait under investigation, although the implications of a slope deviating from 0 are less clear for size than for biomass and photosynthetic activity. For each unique treatment combination (dilution*region*temperature), n=6. The boxplots are displayed as is standard, with the girdle band indicating the median, and the whiskers extending to the 25th and 75th percentile Standard deviations for the slopes can be found in Table S5. Shaded areas in the plot are confidence intervals generated in R and mainly for graphical representation.

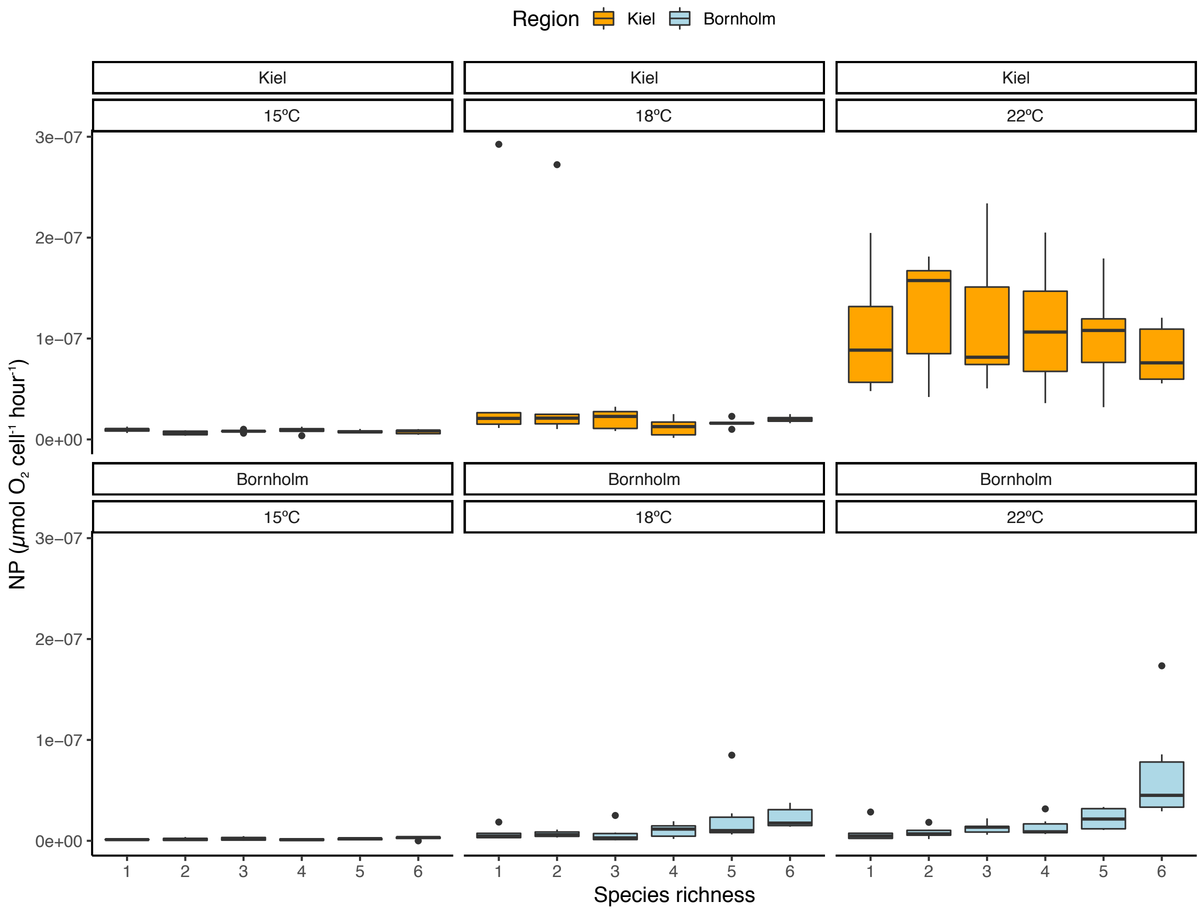

Figure S15 Rates of net photosynthesis (µmol O_2 per cell and hour) **during exponential growth**

During exponential growth, Net Photosynthesis (NP, here in µmol O_2 per cell and hour) in samples from the Kiel sampling stations (orange, upper) and the Bornholm sampling stations (blue, lower) was influenced by assay temperature (individual panels) and dilution (labelled as 'species richness'. Here, displayed as the LOG10 of phytoplankton cells after dilution, which is a good indicator for species richness (see Figure S5 and main manuscript Figure 1). We fitted a slope through LOG10 transformed NP data as this transforms the otherwise exponential relationship into a linear one (see Figure S16). Here, we show the non-transformed data for easier visualisation, as LOG10 transformed data of very small values will be negative. A slope that does not deviate significantly from 0 (see also Table S5) indicates that functional redundancy is high. A slope that does deviate significantly from 0 indicates that species richness has a strong impact on the trait under investigation, with positive slopes for samples with low functional redundancy. While temperature has an impact on net photosynthesis rates in the samples from the Kiel Area (highest rates at 18ºC, lowest at 15ºC, and intermediate values for 22ºC), there is no significant impact of loss of rare species (i.e. dilution). In samples from the Bornholm Basin, NP rates are overall lower, and samples with lower species richness are significantly less photosynthetically active than samples with high species richness, and this trend is exacerbated with increasing temperatures. For each unique treatment combination (dilution*region*temperature), n=6. The boxplots are displayed as is standard, with the girdle band indicating the median, and the whiskers extending to the 25th and 75th percent. Standard deviations for the slopes can be found in Table S5.

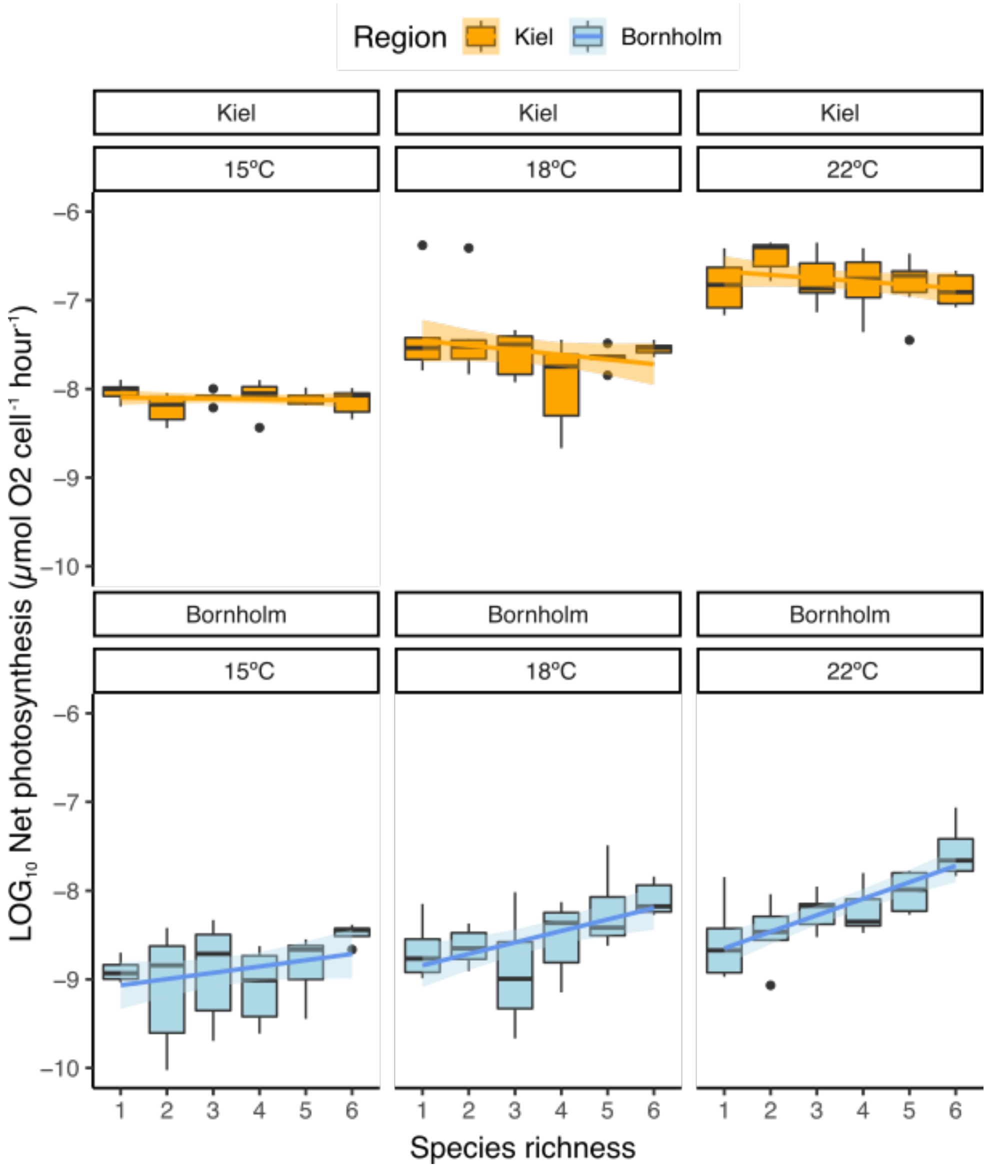

Figure S16 Rates of net photosynthesis (LOG10 µmol O_2 per cell and hour) during exponential growth

This is a LOG10 transformed version of Figure S15 for better visualisation of the slopes. All details are as in Figure S15).

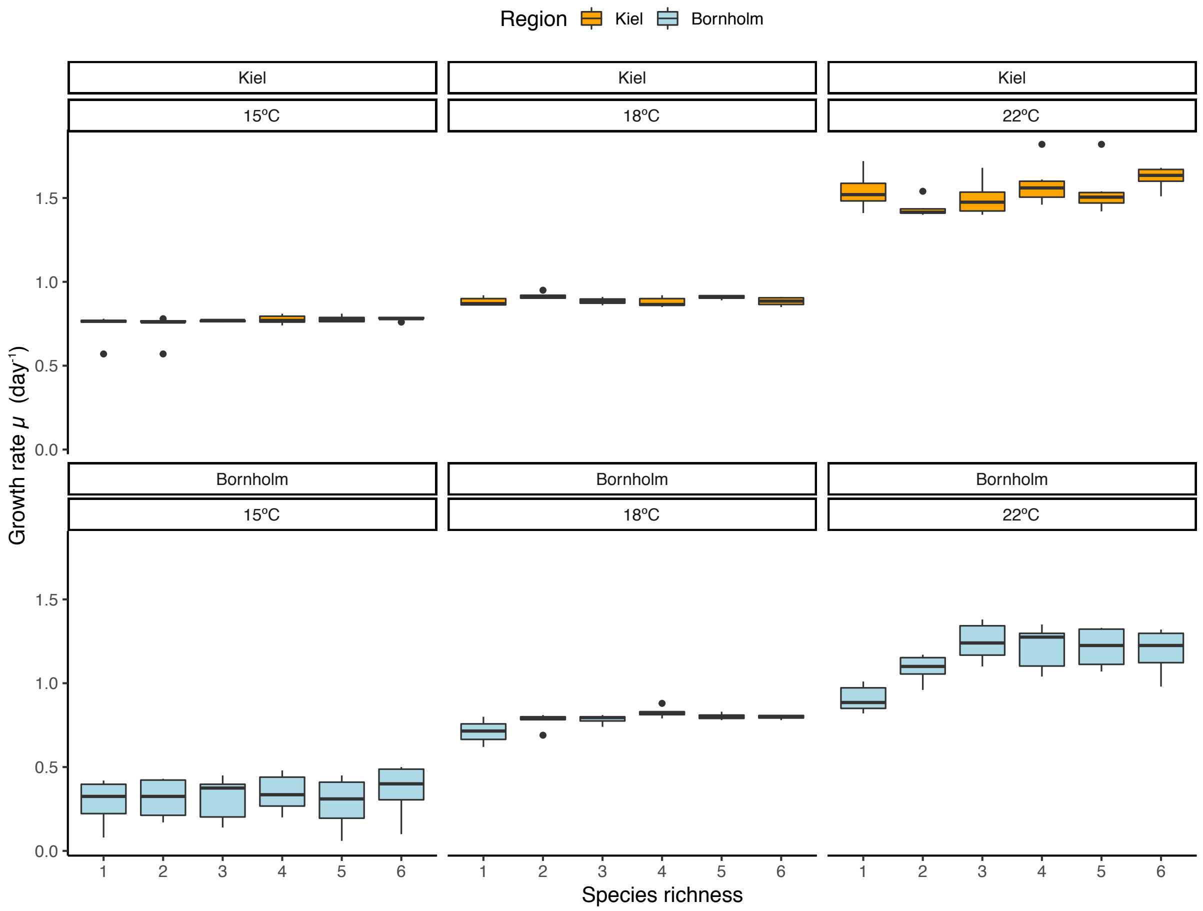

Figure S17: Growth rates (at µmax)

During exponential growth, growth rates in samples from the Kiel sampling stations (orange, upper) and the Bornholm sampling stations (blue, lower) was influenced strongly by geographical origin and assay temperature (individual panels) but only to a smaller degree by dilution (labelled as 'species richness'. Here, displayed as the LOG10 of phytoplankton cells after dilution, which is a good indicator for species richness (see Figure S5 and main manuscript Figure 1). The growth rate values mainly serve to show that experimental units reached the time-points of µmax, and hence carrying capacity, at different points in time and therefore had to be harvested/measured across several days. For each unique treatment combination (dilution*region*temperature), n=6. The boxplots are displayed as is standard, with the girdle band indicating the median, and the whiskers extending to the 25th and 75th percent. We provide the time points at which K was reached at in the data dryad files.

Further Supporting Tables

Table S3: Decomposition analysis for estimates of environmental fluctuations

Random components outcomes produced from decomposition analysis performed on sea surface temperatures time series for Bornholm Basin and the Kiel Area for the last five years, using the function decompose within the anomalize package (0.2.0). We used an additive (seasonal + trend + random) approach, assuming a quarterly seasonality (frequency = 4. The quarterlies are reported in the data dryad tables as Qtr1, Qtr2, Qtr 3 and Qtr4). The table displays the statistical results from a One-way ANOVA comparing the two geographical areas (df: degree of freedom; SS: sum of squares; F: F-value; p: p-value). Mean values for random effect were higher in the Kiel Area, meaning that the time series is less constant and consequently more variable (KA: 1202.95 ± 682.67; BB: 151.94 ± 87.47). No seasonal component was found for the Kiel Area (also using a multiplicative approach assuming a monthly seasonality). We present the detailed quarterlies on data dryad (https://doi.org/10.5061/dryad.0p2ngf1xw). Sea surface temperature monitoring station data for analyses were kindly provided by GEOMAR.

Variable	*df*	SS	*F*	*p*
Geographical Area	1	3.001e+08	718.9	>2e-16

Table S4: Nomenclature for flow cytometry parameters

Name on Accuri C6 display	Name in Accuri C6 file export	As proxy for
FSC	FSC	Size
SSC	SSC	Granularity
FITC	FL1	DNA stain
PE	FL2	Phycoerythrin, Phycocyanin
PerCP	FL3	Chlorophyll a
APC	FL4	Allophyocyanin, Chlorophylls

Table S5: Slopes obtained (per station) from Figures S12 to S16

Slopes for each temperature, region, and station for each trait investigated. Technical replicates have been pooled (to statID), and the slope reported is the average, with 1SD in ‘sd_slope’. NP is for net photosynthesis. NP has been established during exponential growth. Biomass, cell count, and cell size were established during carrying capacity.

Temp	Region	statID	slope	Trait	sd_slope
15	Kiel	1	0.012	Biomass	0.007
15	Kiel	2	0.033	Biomass	0.019
15	Kiel	3	-0.084	Biomass	0.049

15	Bornholm	1	0.005	Biomass	0.003
15	Bornholm	2	-0.032	Biomass	0.019
15	Bornholm	3	-0.011	Biomass	0.006
18	Kiel	1	0.022	Biomass	0.013
18	Kiel	2	0.058	Biomass	0.033
18	Kiel	3	-0.019	Biomass	0.011
18	Bornholm	1	0.087	Biomass	0.05
18	Bornholm	2	0.105	Biomass	0.061
18	Bornholm	3	0.11	Biomass	0.064
22	Kiel	1	0.004	Biomass	0.002
22	Kiel	2	-0.066	Biomass	0.038
22	Kiel	3	-0.012	Biomass	0.007
22	Bornholm	1	0.167	Biomass	0.096
22	Bornholm	2	0.199	Biomass	0.001
22	Bornholm	3	0.184	Biomass	0.106
15	Kiel	1	0	Cell count	0.031
15	Kiel	2	0.022	Cell count	0.025
15	Kiel	3	-0.062	Cell count	0.022
15	Bornholm	1	0.053	Cell count	0.02
15	Bornholm	2	0.108	Cell count	0.018
15	Bornholm	3	0.078	Cell count	0.018
18	Kiel	1	0.019	Cell count	0.021
18	Kiel	2	0.066	Cell count	0.017
18	Kiel	3	0.012	Cell count	0.029
18	Bornholm	1	0.101	Cell count	0.013
18	Bornholm	2	0.102	Cell count	0.024
18	Bornholm	3	0.196	Cell count	0.029
22	Kiel	1	-0.022	Cell count	0.056
22	Kiel	2	0.064	Cell count	0.039
22	Kiel	3	0.017	Cell count	0.033
22	Bornholm	1	0.159	Cell count	0.008
22	Bornholm	2	0.161	Cell count	0.021
22	Bornholm	3	0.167	Cell count	0.046
15	Kiel	1	-0.005	NP	0.009
15	Kiel	2	-0.031	NP	-0.003
15	Kiel	3	-0.001	NP	0.002
15	Bornholm	1	0.076	NP	-0.002
15	Bornholm	2	-0.059	NP	-0.002
15	Bornholm	3	0.147	NP	0
18	Kiel	1	-0.025	NP	0.012
18	Kiel	2	0.007	NP	-0.002
18	Kiel	3	-0.003	NP	-0.002
18	Bornholm	1	0.137	NP	0.003
18	Bornholm	2	0.121	NP	0.006

18	Bornholm	3	0.179	NP	-0.001
22	Kiel	1	-0.001	NP	0.002
22	Kiel	2	0.002	NP	-0.001
22	Kiel	3	-0.009	NP	0
22	Bornholm	1	0.199	NP	0.005
22	Bornholm	2	0.179	NP	0.007
22	Bornholm	3	0.181	NP	0.008
15	Kiel	1	0.001	Size	0
15	Kiel	2	0.001	Size	0
15	Kiel	3	0.002	Size	0
15	Bornholm	1	0.037	Size	0.021
15	Bornholm	2	-0.039	Size	0.023
15	Bornholm	3	0.01	Size	0.006
18	Kiel	1	0.04	Size	0.015
18	Kiel	2	0.004	Size	0.014
18	Kiel	3	0.006	Size	0.014
18	Bornholm	1	-0.668	Size	0.004
18	Bornholm	2	-0.471	Size	0.003
18	Bornholm	3	-0.302	Size	0.001
22	Kiel	1	-0.222	Size	0.003
22	Kiel	2	-0.371	Size	0.002
22	Kiel	3	-0.382	Size	0.002
22	Bornholm	1	-1.387	Size	0.013
22	Bornholm	2	-1.18	Size	0.029
22	Bornholm	3	-1.12	Size	0.018

Table S6: Summary F statistics:

This is a statistics summary (degrees of freedom and F statistics). We recommend that readers look at Tables S7 to S9 in this document for details. In all tables ":" denotes an interaction between factors. Temperature refers to the assay temperature. The F statistics are reported for the model found to be the best model based on AICc scores, not the global model. The denominator DF is lower than the total number of samples minus the number of treatment groups because of the nested nature of the model.

Summary Table 6A: F statistics for Biomass at carrying capacity

	numDF	denDF	F-value	p-value	
(Intercept)	1	102	14566.90	<0.001	***
Region	1	102	182.52	<0.001	***
Temperature	2	102	382.71	<0.001	***
Dilution	5	102	6.93	<0.001	***
Region:Temperature	2	102	42.87	<0.001	***
Region:Dilution	5	102	6.23	<0.001	***
Temperature:Dilution	10	102	4.85	<0.001	***

Region:Temperature:Dilution	10	102	8.96	<0.001	***

Summary Table 6B: F statistics for cell count at carrying capacity

	numDF	denDF	F-value	p-value	
(Intercept)	1	102	14745.38	<0.001	***
Region	1	102	411.09	<0.001	***
Temperature	2	102	184.34	<0.001	***
Dilution	5	102	71.27	<0.001	***
Region:Temperature	2	102	141.63	<0.001	***
Region:Dilution	5	102	45.63	<0.001	***
Temperature:Dilution	10	102	12.67	<0.001	***
Region:Temperature:Dilution	10	102	16.04	<0.001	***

Summary Table 6C: F statistics for cell size at carrying capacity

	numDF	denDF	F-value	p-value	
(Intercept)	1	102	8729.34	<0.001	***
Region	1	102	165.01	<0.001	***
Temperature	2	102	126.97	<0.001	***
Dilution	5	102	11.56	<0.001	***
Region:Temperature	2	102	5.59	0.004	**
Region:Dilution	5	102	5.06	<0.001	***
Temperature: Dilution	10	102	3.26	<0.001	***
Region:Temperature:Dilution	10	102	6.49	<0.001	***

Summary Table 6D: F statistics for net photosynthesis at μmax

	numDF	denDF	F-value	p-value	
(Intercept)	1	102	130.94	<0.001	***
Region	1	102	45.04	<0.001	***
Temperature	2	102	41.78	<0.001	***
Dilution	5	102	1.04	<0.05	*
Region:Temperature	2	102	18.64	<0.001	***

Table S7: Model selection (A) output (B) for investigating the effect of dilution (abbreviated to D), assay temperature (abbreviated T), region (abbreviated R), and season (abbreviated S) on biomass produced at carrying capacity.

In the mixed model, D (from 1 – highest richness to 1e-05 – lowest richness), T (15ºC,18ºC, 22ºC), R (Kiel Area, Bornholm Basin), and S (spring, summer) selection regimes, i.e. nutrient (low nutrient and replete), were fitted as fixed effects. Stations were treated as a random factor. Technical replicates were not fitted. Here and in all other model selection tables, the header indicates the factors considered by a model. When a factor is part of the model, this is shown by a +. When a factor is not considered by a model, this is shown by NA. The best model is highlighted in bold, and is the model with the smallest AICc, where delta AICc to the next best model is >2. By tracing the "+" and "NA" we can see which factors in which combination are or are not part of the model.

df for degrees of freedom; logLik for log likelihood ratio. : indicates an interaction term. We display only the first 10 models for clarity.

The global model formula was lme.formula(K~D*R*S*T, random=~1|bio.stat.id, data=dataframe.K, method="ML) . The model used for the model output table was refitted with REML and read lme.formula(K~D*R*T, random=~1|bio.stat.id, data=dataframe.K, method="REML). In the model output table, CI are the 95% confidence intervals, DF are degrees of freedom. Values other than the first value (Kiel sample at 15ºC with the lowest dilution, i.e. highest diversity) need to be added to the first value to obtain the predicted trait value.

A Intercept	D	T	R	S	D:T	D:R	D:S	T:R	S:T	R:S	D:T:R	D:T:S	D:R:S	T:R:S	D:T:R:S	df	logLik	AICc	Δ AICc	weight
**3.06**	**+**	**+**	**+**	**NA**	**+**	**+**	**NA**	**+**	**NA**	**NA**	**+**	**NA**	**NA**	**NA**	**NA**	**38**	**-129.2**	**339.93**	**0.00**	**0.68**
3.06	+	+	+	+	+	+	NA	+	NA	NA	+	NA	NA	NA	NA	39	-129.2	342.23	2.30	0.22
3.06	+	+	+	+	+	+	NA	+	NA	+	+	NA	NA	NA	NA	40	-129.2	344.53	4.60	0.07
3.06	+	+	+	+	+	+	NA	+	+	NA	+	NA	NA	NA	NA	41	-129.2	346.75	6.82	0.02
3.06	+	+	+	+	+	+	NA	+	+	+	+	NA	NA	NA	NA	42	-129.2	349.07	9.14	0.01
3.06	+	+	+	+	+	+	+	+	NA	NA	+	NA	NA	NA	NA	44	-128.6	352.67	12.74	0.00
3.06	+	+	+	+	+	+	NA	+	+	+	+	NA	NA	+	NA	44	-129	353.51	13.58	0.00
3.06	+	+	+	+	+	+	+	+	NA	+	+	NA	NA	NA	NA	45	-128.6	355.01	15.09	0.00
3.06	+	+	+	+	+	+	+	+	+	NA	+	NA	NA	NA	NA	46	-128.6	357.29	17.36	0.00
3.06	+	+	+	+	+	+	+	+	+	+	+	NA	NA	NA	NA	47	-128.6	359.65	19.72	0.00

B	Value	CI (lower)	CI (upper)	Std.Error	DF	t-value	p-value	
Region:Kiel (at 15 C, least dilute)	3.06	2.90	3.21	0.08	13	38.24	<0.001	***
Region: Bornholm (at 15 C, least dilute)	0.54	0.32	0.76	0.11	13	4.81	<0.001	***
Temp18	0.42	0.21	0.64	0.11	13	3.89	<0.001	***
Temp22	1.05	0.82	1.28	0.12	13	8.96	<0.001	***
Dilution1e-05	0.09	-0.13	0.31	0.11	13	0.77	0.441	
Dilution1e-04	0.10	-0.11	0.30	0.11	13	0.89	0.374	
Dilution0.001	0.19	-0.02	0.39	0.11	13	1.76	0.079	.
Dilution0.01	-0.01	-0.21	0.20	0.11	13	-0.07	0.942	
Dilution0.1	-0.09	-0.30	0.12	0.11	13	-0.82	0.41	
Region: Bornholm: Temp18	-0.70	-1.00	-0.39	0.16	13	-4.51	<0.001	***
Region: Bornholm: Temp22	-1.81	-2.14	-1.48	0.17	13	-10.78	<0.001	***
Region: Bornholm: Dilution1e-05	-0.32	-0.64	0.00	0.16	13	-1.95	0.052	.
Region: Bornholm: Dilution1e-04	-0.22	-0.53	0.09	0.16	13	-1.40	0.161	
Region: Bornholm: Dilution0.001	-0.34	-0.66	-0.03	0.16	13	-2.16	0.031	*
Region: Bornholm: Dilution0.01	-0.37	-0.68	-0.06	0.16	13	-2.37	0.018	*
Region: Bornholm: Dilution0.1	-0.22	-0.53	0.09	0.16	13	-1.38	0.169	
Temp18: Dilution1e-05	-0.18	-0.49	0.13	0.16	13	-1.16	0.248	
Temp22: Dilution1e-05	-0.17	-0.50	0.15	0.16	13	-1.05	0.296	
Temp18: Dilution1e-04	-0.07	-0.37	0.22	0.15	13	-0.49	0.623	
Temp22: Dilution1e-04	-0.41	-0.73	-0.09	0.16	13	-2.55	0.011	*
Temp18: Dilution0.001	0.05	-0.25	0.35	0.15	13	0.31	0.759	
Temp22: Dilution0.001	-0.39	-0.70	-0.08	0.16	13	-2.43	0.015	*
Temp18: Dilution0.01	0.15	-0.15	0.44	0.15	13	0.98	0.327	
Temp22: Dilution0.01	-0.18	-0.50	0.13	0.16	13	-1.15	0.25	
Temp18: Dilution0.1	0.05	-0.25	0.35	0.15	13	0.34	0.731	

Temp22: Dilution0.1	-0.06	-0.38	0.25	0.16	13	-0.39	0.694	
Region: Bornholm: Temp18: Dilution1e-05	0.70	0.26	1.13	0.22	13	3.14	0.002	**
Region: Bornholm: Temp22: Dilution1e-05	0.90	0.44	1.37	0.24	13	3.82	<0.001	***
Region: Bornholm: Temp18: Dilution1e-04	0.46	0.03	0.89	0.22	13	2.12	0.034	*
Region: Bornholm: Temp22: Dilution1e-04	1.43	0.98	1.88	0.23	13	6.22	<0.001	***
Region: Bornholm: Temp18: Dilution0.001	0.40	-0.04	0.83	0.22	13	1.80	0.072	.
Region: Bornholm: Temp22: Dilution0.001	1.73	1.28	2.19	0.23	13	7.54	<0.001	***
Region: Bornholm: Temp18: Dilution0.01	0.69	0.27	1.11	0.22	13	3.20	0.001	**
Region: Bornholm: Temp22: Dilution0.01	1.49	1.04	1.94	0.23	13	6.53	<0.001	***
Region: Bornholm: Temp18: Dilution0.1	0.63	0.20	1.06	0.22	13	2.87	0.004	**
Region: Bornholm: Temp22: Dilution0.1	1.43	0.98	1.88	0.23	13	6.23	<0.001	***

Table S8: Model selection (A) output (B) for investigating the effect of dilution (abbreviated to D), assay temperature (abbreviated T), region (abbreviated R), and season (abbreviated S) on cell diameter (µm) at carrying capacity.
In the mixed model, D (from 1 – highest richness to 1e-05 – lowest richness), T (15ºC,18ºC, 22ºC), R (Kiel Area, Bornholm Basin), and S (spring, summer) selection regimes, i.e. nutrient (low nutrient and replete), were fitted as fixed effects. Stations were treated as a random factor. Technical replicates were not fitted. Here and in all other model selection tables, the header indicates the factors considered by a model. When a factor is part of the model, this is shown by a +. When a factor is not considered by a model, this is shown by NA. The best model is highlighted in bold, and is the model with the smallest AICc, where delta AICc to the next best model is >2. By tracing the "+" and "NA" we can see which factors in which combination are or are not part of the model.
df for degrees of freedom; logLik for log likelihood ratio. : indicates an interaction term. We display only the first 10 models for clarity.
The global model formula was lme.formula(sizeum~D*R*S*T, random=~1|bio.stat.id, data=dataframe.sizeum, method="ML) . The model used for the model output table was refitted with REML and read lme.formula(sizeum ~D*R*T, random=~1|bio.stat.id, data=dataframe. sizeum, method="REML). In the model output table, CI are the 95% confidence intervals, DF are degrees of freedom. Values other than the first value (Kiel sample at 15ºC with the lowest dilution, i.e. highest diversity) need to be added to the first value to obtain the predicted trait value.

A) Intercept	D	T	R	S	D:T	D:R	D:S	T:R	S:T	R:S	D:T:R	D:T:S	D:R:S	T:R:S	D:T:R:S	df	logLik	AICc	Δ	weight
**2.92**	**+**	**+**	**+**	**NA**	**+**	**+**	**NA**	**+**	**NA**	**NA**	**+**	**NA**	**NA**	**NA**	**NA**	**38**	**-863.16**	**1807.83**	**0**	**0.65**
2.9	+	+	+	+	+	+	NA	+	NA	NA	+	NA	NA	NA	NA	39	-863.06	1809.92	2.1	0.23
2.9	+	+	+	+	+	+	NA	+	NA	+	+	NA	NA	NA	NA	40	-863.03	1812.18	4.35	0.07
2.96	+	+	+	+	+	+	NA	+	+	NA	+	NA	NA	NA	NA	41	-862.61	1813.66	5.83	0.03
2.95	+	+	+	+	+	+	NA	+	+	+	+	NA	NA	NA	NA	42	-862.57	1815.91	8.08	0.01
2.93	+	+	+	+	+	+	NA	+	+	+	+	NA	NA	+	NA	44	-861.26	1817.97	10.14	0
2.89	+	+	+	+	+	+	+	+	NA	NA	+	NA	NA	NA	NA	44	-862.29	1820.02	12.19	0
2.88	+	+	+	+	+	+	+	+	NA	+	+	NA	NA	NA	NA	45	-862.26	1822.32	14.49	0
2.94	+	+	+	+	+	+	+	+	+	NA	+	NA	NA	NA	NA	46	-861.83	1823.82	15.99	0
2.93	+	+	+	+	+	+	+	+	+	+	+	NA	NA	NA	NA	47	-861.79	1826.12	18.29	0

B)	Value	CI (lower)	CI (upper)	Std.Error	DF	t-value	p-value	
Region:Kiel (at 15 C, least dilute)	2.92	3.39	4.46	0.27	13	14.42	<0.001	***
Region: Bornholm (at 15 C, least dilute)	1.14	1.35	2.93	0.40	13	5.33	<0.001	***
Temp18	0.73	-0.04	1.49	0.39	13	1.87	0.062	.
Temp22	1.17	1.34	3.00	0.42	13	5.16	<0.001	***
Dilution1e-05	0.75	-0.04	1.54	0.40	13	1.86	0.064	.
Dilution1e-04	0.40	-0.35	1.15	0.38	13	1.06	0.292	
Dilution0.001	0.17	-0.57	0.92	0.38	13	0.46	0.643	
Dilution0.01	0.15	-0.59	0.89	0.38	13	0.40	0.687	
Dilution0.1	0.42	-0.34	1.19	0.39	13	1.09	0.275	
Region: Bornholm: Temp18	-0.01	-1.10	1.08	0.56	13	-0.02	0.988	
Region: Bornholm: Temp22	-2.53	-3.71	-1.35	0.60	13	-4.21	<0.001	***
Region: Bornholm: Dilution1e-05	-0.75	-1.89	0.40	0.58	13	-1.28	0.2	
Region: Bornholm: Dilution1e-04	-0.25	-1.35	0.86	0.56	13	-0.44	0.662	
Region: Bornholm: Dilution0.001	-0.36	-1.48	0.76	0.57	13	-0.63	0.527	
Region: Bornholm: Dilution0.01	-2.73	-3.83	-1.63	0.56	13	-4.87	<0.001	***
Region: Bornholm: Dilution0.1	-3.30	-4.41	-2.18	0.57	13	-5.81	<0.001	***
Temp18: Dilution1e-05	-0.73	-1.83	0.37	0.56	13	-1.31	0.191	
Temp22: Dilution1e-05	-1.69	-2.85	-0.54	0.59	13	-2.87	0.004	**
Temp18: Dilution1e-04	-0.63	-1.69	0.44	0.54	13	-1.16	0.247	
Temp22: Dilution1e-04	-1.46	-2.60	-0.33	0.58	13	-2.54	0.011	*
Temp18: Dilution0.001	-0.57	-1.64	0.50	0.55	13	-1.04	0.299	
Temp22: Dilution0.001	-1.36	-2.49	-0.24	0.57	13	-2.38	0.018	*
Temp18: Dilution0.01	-0.48	-1.54	0.57	0.54	13	-0.90	0.368	
Temp22: Dilution0.01	-1.40	-2.53	-0.27	0.57	13	-2.44	0.015	*
Temp18: Dilution0.1	-0.47	-1.54	0.60	0.55	13	-0.87	0.387	

Temp22: Dilution0.1	-2.10	-3.24	-0.96	0.58	13	-3.62	<0.001	***
Region: Bornholm: Temp18: Dilution1e-05	0.73	-0.82	2.29	0.79	13	0.92	0.356	
Region: Bornholm: Temp22: Dilution1e-05	1.69	0.03	3.36	0.85	13	2.00	0.046	*
Region: Bornholm: Temp18: Dilution1e-04	0.10	-1.43	1.62	0.78	13	0.12	0.903	
Region: Bornholm: Temp22: Dilution1e-04	1.43	-0.18	3.04	0.82	13	1.74	0.083	.
Region: Bornholm: Temp18: Dilution0.001	-0.37	-1.92	1.19	0.79	13	-0.46	0.643	
Region: Bornholm: Temp22: Dilution0.001	1.29	0.67	3.90	0.82	13	2.78	0.006	**
Region: Bornholm: Temp18: Dilution0.01	1.66	0.14	3.18	0.77	13	2.14	0.032	*
Region: Bornholm: Temp22: Dilution0.01	3.05	2.45	5.65	0.82	13	4.97	<0.001	***
Region: Bornholm: Temp18: Dilution0.1	1.85	0.30	3.40	0.79	13	2.35	0.019	*
Region: Bornholm: Temp22: Dilution0.1	3.45	3.84	7.06	0.82	13	6.65	<0.001	***

Table S9: Model selection (A) output (B) for investigating the effect of dilution (abbreviated to D), assay temperature (abbreviated T), region (abbreviated R), and season (abbreviated S) on Net Photosynthesis rates (µmol O_2 per cell and hour - displayed as LOG10 values for brevity) during exponential growth.

In the mixed model, D (from 1 – highest richness to 100000– lowest richness), T (15ºC,18ºC, 22ºC), R (Kiel Area, Bornholm Basin), and S (spring, summer) selection regimes, i.e. nutrient (low nutrient and replete), were fitted as fixed effects. Stations were treated as a random factor. Technical replicates were not fitted. Here and in all other model selection tables, the header indicates the factors considered by a model. When a factor is part of the model, this is shown by a +. When a factor is not considered by a model, this is shown by NA. The best model is highlighted in bold, and is the model with the smallest AICc, where delta AICc to the next best model is >2. By tracing the "+" and "NA" we can see which factors in which combination are or are not part of the model.

df for degrees of freedom; logLik for log likelihood ratio. : indicates an interaction term. We display only the first 10 models for clarity.

The global model formula was lme.formula(sizeum~D*R*S*T, random=~1|bio.stat.id, data=dataframe.sizeum, method="ML) . The model used for the model output table was refitted with REML and read lme.formula(sizeum ~R*T +D, random=~1|bio.stat.id, data=dataframe. sizeum, method="REML). In the model output table, CI are the 95% confidence intervals, DF are degrees of freedom. Values other than the first value (Kiel sample at 15ºC with the lowest dilution, i.e. highest diversity) need to be added to the first value to obtain the predicted trait value.

Inter-cept	T	D	R	S	T*D	T*R	T*S	D*R	D*S	R*S	T*D*R	T*D*S	T*R*S	D*R*S	T*D*R*S	df	logLik	AICc	delta	weight
**-7.02E-09**	**+**	**+**	**+**	**NA**	**NA**	**+**	**NA**	**+**	**NA**	**NA**	**NA**	**NA**	**NA**	**NA**	**NA**	**18**	**3397.38**	**-6755.28**	**0.00**	**0.36**
-7.01E-09	+	+	+	+	NA	+	+	+	NA	NA	NA	NA	NA	NA	NA	21	3400.32	-6757.35	2.08	0.18
-6.93E-09	+	+	+	+	NA	+	NA	+	NA	NA	NA	NA	NA	NA	NA	19	3397.38	-6752.87	2.40	0.11
8.07E-09	+	NA	+	NA	NA	+	NA	NA	NA	NA	NA	NA	NA	NA	NA	8	3384.71	-6752.72	2.56	0.10
-6.19E-09	+	+	+	+	NA	+	+	+	NA	+	NA	NA	NA	NA	NA	22	3400.37	-6751.51	3.77	0.05
8.08E-09	+	NA	+	+	NA	+	+	NA	NA	NA	NA	NA	NA	NA	NA	11	3387.32	-6751.35	3.93	0.05
-6.12E-09	+	+	+	+	NA	+	NA	+	NA	+	NA	NA	NA	NA	NA	20	3397.43	-6750.56	4.72	0.03
8.16E-09	+	NA	+	+	NA	+	NA	NA	NA	NA	NA	NA	NA	NA	NA	9	3384.71	-6750.55	4.73	0.03
-6.99E-09	+	+	+	+	NA	+	+	+	NA	+	NA	NA	+	NA	NA	24	3402.21	-6750.14	5.14	0.03
8.90E-09	+	NA	+	+	NA	+	+	NA	NA	+	NA	NA	NA	NA	NA	12	3387.37	-6749.21	6.07	0.02
8.97E-09	+	NA	+	+	NA	+	NA	NA	NA	+	NA	NA	NA	NA	NA	10	3384.76	-6748.45	6.83	0.01

B)	Value	CI (lower)	CI (upper)	Std.Error	DF	t-value	p-value	
Region:Kiel (at 15 C, least dilute)	1.00E-08	-7.07E-09	2.71E-08	8.66E-09	200	1.16	0.249	
Region: Bornholm (at 15 C, least dilute)	-6.28E-09	-2.41E-08	1.16E-08	9.05E-09	200	-0.69	0.489	
Temp18	2.43E-08	6.44E-09	4.21E-08	9.05E-09	200	2.68	0.008	**
Temp22	9.39E-08	7.60E-08	1.12E-07	9.05E-09	200	10.37	<0.001	***
Dilution10	-5.12E-09	-2.30E-08	1.27E-08	9.05E-09	200	-0.57	0.572	
Dilution100	-7.96E-09	-2.58E-08	9.88E-09	9.05E-09	200	-0.88	0.38	
Dilution1000	-6.64E-09	-2.45E-08	1.12E-08	9.05E-09	200	-0.73	0.464	
Dilution10000	9.95E-09	-7.90E-09	2.78E-08	9.05E-09	200	1.10	0.273	
Dilution100000	-1.89E-09	-1.97E-08	1.60E-08	9.05E-09	200	-0.21	0.835	
Region: Bornholm: Temp18	-1.31E-08	-3.83E-08	1.22E-08	1.28E-08	200	-1.02	0.308	
Region: Bornholm:Temp22	-7.33E-08	-9.86E-08	-4.81E-08	1.28E-08	200	-5.73	<0.001	***

Supporting References

1. Keller, M. D., Selvin, R. C., Claus, W. & Guillard, R. R. L. 1987 Media for the Culture of Oceanic Ultraphytoplankton. *Journal of Phycology* **23**, 633–638. (doi:10.1111/j.1529-8817.1987.tb04217.x)

2. Angilletta, M. J., Jr, Wilson, R. S., Navas, C. A. & James, R. S. 2003 Tradeoffs and the evolution of thermal reaction norms. *Trends in Ecology & Evolution* **18**, 234–240. (doi:10.1016/S0169-5347(03)00087-9)

3. Kawakami, T., Morgan, T. J., Nippert, J. B., Ocheltree, T. W., Keith, R., Dhakal, P. & Ungerer, M. C. 2011 Natural Selection Drives Clinal Life History Patterns In The Perennial Sunflower Species, Helianthus Maximiliani. *Molecular Ecology* **20**, 2318–2328. (doi:10.1111/j.1365-294X.2011.05105.x)

4. de Villemereuil, P., Gaggiotti, O. E., Mouterde, M. & Till-Bottraud, I. 2016 Common garden experiments in the genomic era: new perspectives and opportunities. *Heredity* **116**, 249–254.

5. Fontana, S., Thomas, M. K., Moldoveanu, M., Spaak, P. & Pomati, F. 2018 Individual-level trait diversity predicts phytoplankton community properties better than species richness or evenness. *The ISME Journal* **12**, 356–366.

6. García, F. C., Alonso-Sáez, L., Morán, X. A. G. & López-Urrutia, Á. 2015 Seasonality in molecular and cytometric diversity of marine bacterioplankton: the re-shuffling of bacterial taxa by vertical mixing. *Environmental Microbiology* **17**, 4133–4142. (doi:10.1111/1462-2920.12984)

7. Props, R., Monsieurs, P., Mysara, M., Clement, L. & Boon, N. 2016 Measuring the biodiversity of microbial communities by flow cytometry. *Methods Ecol Evol* **7**, 1376–1385. (doi:10.1111/2041-210X.12607)

8. Montagnes, D. J. S., Berges, J. A., Harrison, P. J. & Taylor, F. J. R. 1994 Estimating carbon, nitrogen, protein, and chlorophyll a from volume in marine phytoplankton. *Limnol. Oceanogr.* **39**, 1044–1060. (doi:10.4319/lo.1994.39.5.1044)

9. Fawley, M. W., Phycology, K. F. J. O.2004 In press. A Simple And Rapid Technique For The Isolation Of Dna From Microalgae1. *Wiley Online Library* **40**, 223–225. (doi:10.1046/j.1529-8817.2004.03081.x)

10. Rose, J. M., Caron, D. A., Sieracki, M. E. & Poulton, N. 2004 Counting heterotrophic nanoplanktonic protists in cultures and aquatic communities by flow cytometry. *Aquat. Microb. Ecol.* **34**, 263–277.

11. Buchanan, R. L., Whiting, R. C. & Damert, W. C. 1997 When is simple good enough: a comparison of the Gompertz, Baranyi, and three-phase linear models for fitting bacterial growth curves. *Food Microbiology* **14**, 313–326. (doi:10.1006/fmic.1997.0125)

12. Murphy, J. & Riley, J. P. 1962 A modified single solution method for the determination of phosphate in natural waters. *Analytica Chimica Acta* **27**, 31–36. (doi:10.1016/s0003-2670(00)88444-5)

13. Grasshoff, K., Kremling, K. & Ehrhardt, M. 2009 *Methods of seawater analysis*.

References for R packages used

Kamil Bartoń (2019). **MuMIn**: Multi-Model Inference. R package version 1.43.6. https://CRAN.R-project.org/package=MuMIn

F. Baty and M. L. Delignette-Muller (2013), **nlsMicrobio**: data sets and nonlinear regression models dedicated to predictive microbiology.

Pinheiro J, Bates D, DebRoy S, Sarkar D, R Core Team (2019). _**nlme**: Linear and Nonlinear Mixed Effects Models_. R package version 3.1-140, <URL: https://CRAN.R-project.org/package=nlme>.

B. Hofner (2019). **papeR**: A Toolbox for Writing Pretty Papers and Reports, R package version 1.0-4, https://CRAN.R-project.org/package=papeR.

Jari Oksanen, F. Guillaume Blanchet, Michael Friendly, Roeland Kindt, Pierre Legendre, Dan McGlinn, Peter R. Minchin, R. B. O'Hara, Gavin L. Simpson, Peter Solymos, M. Henry H. Stevens, Eduard Szoecs and Helene Wagner (2019). **vegan**: Community Ecology Package. R package version 2.5-6. https://CRAN.R-project.org/package=vegan

Props et al. 2016, **phenoFlow:** has no in-R citation, but the package is available here https://github.com/rprops/Phenoflow_package/wiki/1.-Phenotypic-diversity-analysis , and the accompanying manuscript is [7]

H. Wickham. **ggplot2**: Elegant Graphics for Data Analysis. Springer-Verlag New York, 2016.

Hadley Wickham, Jim Hester and Winston Chang (2019). **devtools**: Tools to Make Developing R Packages Easier. R package version 2.1.0. https://CRAN.R-project.org/package=devtools

Claus O. Wilke (2019). **cowplot**: Streamlined Plot Theme and Plot Annotations for 'ggplot2'. R package version 0.9.4. https://CRAN.R-project.org/package=cowplot
